# APOE4 exacerbates α-synuclein seeding activity and contributes to neurotoxicity in Alzheimer’s disease with Lewy body pathology

**DOI:** 10.1007/s00401-022-02421-8

**Published:** 2022-04-26

**Authors:** Yunjung Jin, Fuyao Li, Berkiye Sonoustoun, Naveen Chandra Kondru, Yuka A. Martens, Wenhui Qiao, Michael G. Heckman, Tadafumi C. Ikezu, Zonghua Li, Jeremy D. Burgess, Danilyn Amerna, Justin O’Leary, Michael A. DeTure, Jing Zhao, Pamela J. McLean, Dennis W. Dickson, Owen A. Ross, Guojun Bu, Na Zhao

**Affiliations:** 1 Department of Neuroscience, Mayo Clinic, 4500 San Pablo Road, Jacksonville, FL 32224 USA; 2 Division of Biomedical Statistics and Informatics, Mayo Clinic, Jacksonville, FL 32224 USA

**Keywords:** Alzheimer’s disease, Lewy body dementia, Apolipoprotein E, α-synuclein, Seeding, RT-QuIC

## Abstract

Approximately half of Alzheimer’s disease (AD) brains have concomitant Lewy pathology at autopsy, suggesting that α-synuclein (α-SYN) aggregation is a regulated event in the pathogenesis of AD. Genome-wide association studies revealed that the ε4 allele of the apolipoprotein E (*APOE4*) gene, the strongest genetic risk factor for AD, is also the most replicated genetic risk factor for Lewy body dementia (LBD), signifying an important role of APOE4 in both amyloid-β (Aβ) and α-SYN pathogenesis. How APOE4 modulates α-SYN aggregation in AD is unclear. In this study, we aimed to determine how α-SYN is associated with AD-related pathology and how APOE4 impacts α-SYN seeding and toxicity. We measured α-SYN levels and their association with other established AD-related markers in brain samples from autopsy-confirmed AD patients (*N* = 469), where 54% had concomitant LB pathology (AD + LB). We found significant correlations between the levels of α-SYN and those of Aβ40, Aβ42, tau and APOE, particularly in insoluble fractions of AD + LB. Using a real-time quaking-induced conversion (RT-QuIC) assay, we measured the seeding activity of soluble α-SYN and found that α-SYN seeding was exacerbated by APOE4 in the AD cohort, as well as a small cohort of autopsy-confirmed LBD brains with minimal Alzheimer type pathology. We further fractionated the soluble AD brain lysates by size exclusion chromatography (SEC) ran on fast protein liquid chromatography (FPLC) and identified the α-SYN species (~ 96 kDa) that showed the strongest seeding activity. Finally, using human induced pluripotent stem cell (iPSC)-derived neurons, we showed that amplified α-SYN aggregates from AD + LB brain of patients with *APOE4* were highly toxic to neurons, whereas the same amount of α-SYN monomer was not toxic. Our findings suggest that the presence of LB pathology correlates with AD-related pathologies and that APOE4 exacerbates α-SYN seeding activity and neurotoxicity, providing mechanistic insight into how APOE4 affects α-SYN pathogenesis in AD.

**Supplementary Information:**

The online version contains supplementary material available at 10.1007/s00401-022-02421-8.

## Introduction

Alzheimer’s disease (AD) is a neurodegenerative disorder defined by the presence of two pathological features: amyloid-β (Aβ) plaques and tau neurofibrillary tangles [12]. AD brains often contain additional pathological lesions, including Lewy-related pathology and TAR DNA-binding protein 43 (TDP-43) pathology [29, 45, 51, 61]. Notably, Lewy-related pathology, including Lewy bodies (LB) and Lewy neurites (LN), has been reported in more than 50% of autopsy-confirmed AD cases [4, 13, 14, 43, 47]. The primary constituent of Lewy-related pathology is α-synuclein (α-SYN). Lewy-related pathology is the defining feature of Lewy body dementia (LBD), which includes Parkinson’s disease dementia (PDD) and dementia with Lewy bodies (DLB) [23, 32]. There is limited understanding of the mechanisms that lead to aggregation of α-SYN and its related toxicity in AD.

Apolipoprotein E (APOE), which has three isoforms in humans (APOE2, APOE3 and APOE4), plays key role in lipid transport and metabolism by delivering lipids to neurons in support of brain functions [66, 71]. The *APOE4* gene allele is the strongest genetic risk factor for sporadic AD [71]. APOE4 impacts amyloid during the seeding stage by perturbing Aβ clearance and enhancing Aβ aggregation [22, 66]. Interestingly, a genome-wide association study (GWAS) of LBD revealed the top hit to be *APOE4* (OR 2.46; *p* = 3.31 × 10^–64^) [21], although the effect is less than that seen in AD (OR 3.32; *p* = 1.2 × 10^–881^) [34]. These results suggest that AD and LBD have common or overlapping pathogenic mechanisms. Previous studies have reported that *APOE4* is associated with increased α-SYN pathology in LBD brains with minimal AD pathology [13, 59]. Supporting this observation, we have generated AAV-mediated synucleinopathy animal models expressing APOE isoforms and found increased α-SYN pathology and neurodegeneration in human *APOE4* knock-in mice overexpressing α-SYN, but minimal changes in *APOE2* or *APOE3* mice [70]. Together, these results support a direct effect of *APOE4* on α-SYN pathology and toxicity. How the APOE4 protein isoform modifies α-SYN aggregation in humans in the presence of Aβ has not been addressed.

Herein, we performed biochemical analyses measuring levels of α-SYN and other key AD-related molecules in autopsy-confirmed AD cases (*N* = 469) from the Mayo Clinic brain bank; 54% of the cases had AD with Lewy body pathology (AD + LB). The cohort was further divided into *APOE4* non-carriers (*APOE4*^*−*^) and carriers (*APOE4*^+^). We measured by ELISA total α-SYN in both soluble and insoluble fractions of brain lysates from the superior temporal cortex, one of the most vulnerable neocortical regions to Lewy-related pathology. The α-SYN levels in different fractions were compared in different subgroups, and the association of α-SYN levels were correlated with markers of AD pathology (including Aβ40, Aβ42, tau and APOE). Recent studies have established the α-SYN real-time quaking-induced conversion (RT-QuIC) assay as a sensitive method to evaluate the seeding potential of α-SYN in biological samples, including brains, biofluids, peripheral tissues and cell cultures [40, 42]. Therefore, we determined the seeding activity of α-SYN using RT-QuIC assay in soluble TBS protein fractions of brain lysates. We further fractionated the soluble brain lysates by size exclusion chromatography (SEC) ran on fast protein liquid chromatography (FPLC) to identify the α-SYN species that are responsible for seeding activity. Finally, using human induced pluripotent stem cells (iPSC)-derived neurons, we tested the neuronal toxicity of the amplified α-SYN aggregates from these human brains.

## Materials and methods

### Human postmortem brain sample preparation

This study was conducted in accordance with a protocol approved by the Mayo Clinic Institutional Review Board. A total of 469 autopsy-confirmed AD cases were obtained from the brain bank for neurodegenerative disorders at Mayo Clinic in Jacksonville as previously described [35]. All cases were non-Hispanic white decedents and had undergone standardized neuropathologic sampling and evaluation as described previously [53]. Briefly, thioflavin-S fluorescent microscopy was used to evaluate AD neuropathologic change, including Braak tangle stage and Thal amyloid phase [7, 57]. Lewy pathology was assessed with α-SYN immunohistochemistry and the Lewy bodies were counted in the cingulate, inferior parietal, middle frontal, parahippocampal and superior temporal gyri, as well as in the substantia nigra [13]. The cases were classified as brainstem (BLBD), transitional (TLBD), diffuse (DLBD) Lewy body disease, or amygdala predominant Lewy body disease (ALB) according to the criteria proposed by the International Consortium for Lewy Body Dementia [43]. Thioflavin-S microscopy was also used to assess cerebral amyloid angiopathy (CAA) severity in the superior temporal cortex, inferior parietal cortex, middle frontal cortex, motor cortex, and visual cortex using a semi-quantitative method as follows: 0, no amyloid positive vessels; 0.5, Amyloid deposition restricted to the leptomeninges; 1, mild amyloid deposition observed in the leptomeninges and parenchymal vessels or parenchymal vessels only; 2, moderate circumferential amyloid deposition in some vessels; 3, widespread severe amyloid deposition in leptomeninges and parenchymal vessels; 4, even more severe CAA with dyshoric changes noted (only one case in this cohort) [65]. The averaged CAA scores were quantified from these five cortical regions. Genotyping for *APOE* single nucleotide variants (rs429358 C/T and rs7412 C/T), which define the *APOE2*, *APOE3*, and *APOE4* alleles, was performed using custom TaqMan Allelic Discrimination Assays on a QuantStudio 7 Flex Real-Time PCR system (Applied Bio-Systems). A relatively smaller cohort of autopsy-confirmed 16 control brains and 18 LBD brains with minimal Alzheimer type pathology (Braak stages 0 to III and Thal phases 0 to 1) were also included for testing α-SYN seeding. These cases were selected from the same brain bank with a known *APOE* genotype. The *APOE4*^+^ cases from each group were included first and then the age- and sex-matched *APOE4*^*−*^ cases were selected. The sample characteristics of these cohorts are summarized in Tables 1 and 2, and Supplementary Table 1 (online resource).

**Table 1:** Patient characteristics for AD cohorts

Variable	AD without LB pathology (AD, *N* = 214)		AD with LB pathology (AD + LB, N = 255)	
	*APOE4* non-carrier (*N* = 80)	*APOE4* carrier (*N* = 134)	*APOE4* non-carrier (*N* = 78)	*APOE4* carrier (*N* = 177)
Age (years)	81.0 (55.0, 96.0)	83.0 (55.0, 100.0)	78.0 (57.0, 98.0)	81.0 (58.0, 99.0)
Sex (male)	38 (47.5%)	61 (45.5%)	41 (52.6%)	84 (47.5%)
*Thal phase*				
2	1 (1.2%)	0 (0.0%)	1 (1.3%)	1 (0.6%)
3	8 (10.0%)	10 (7.5%)	9 (11.7%)	5 (2.8%)
4	6 (7.5%)	10 (7.5%)	4 (5.2%)	17 (9.6%)
5	65 (81.2%)	114 (85.1%)	63 (81.8%)	154 (87.0%)
*Braak stage*				
IV	10 (12.5%)	23 (17.2%)	13 (16.7%)	25 (14.1%)
V	33 (41.2%)	36 (26.9%)	27 (34.6%)	47 (26.6%)
VI	37 (46.2%)	75 (56.0%)	38 (48.7%)	105 (59.3%)

The sample median (minimum, maximum) is given for age

**Table 2:** Patient characteristics for control and pure LBD cases

Variable	Control (*N* = 16)		LBD (*N* = 18)	
	*APOE4* non-carrier (*N* = 8)	*APOE4* carrier (*N* = 8)	*APOE4* non-carrier (*N* = 9)	*APOE4* carrier (*N* = 9)
Age (years)	82.5 (79.0, 91.0)	87.0 (61.0, 97.0)	70.0 (61.0, 83.0)	72.0 (65.0, 82.0)
Sex (male)	4 (50.0%)	5 (62.5%)	8 (88.9%)	8 (88.9%)
*Thal phase*				
0	4 (50.0%)	1 (12.5%)	6 (1.3%)	2 (0.6%)
1	1 (12.5%)	1 (12.5%)	3 (11.7%)	7 (2.8%)
2	3 (37.5%)	2 (25.0%)	0 (5.2%)	0 (9.6%)
3	0 (0.0%)	4 (50.0%)	0 (81.8%)	0 (87.0%)
*Braak stage*				
0	2 (25.0%)	2 (25.0%)	1 (11.1%)	3 (33.3%)
I	3 (37.5%)	2 (25.0%)	2 (22.2%)	1 (11.1%)
II	2 (25.0%)	3 (37.5%)	6 (66.7%)	2 (22.2%)
III	1 (12.5%)	1 (12.5%)	0 (0.0%)	3 (33.3%)

The sample median (minimum, maximum) is given for age

Samples of frozen tissue from the superior temporal cortex were subjected to three-step sequential protein extractions using Tris-buffered saline (TBS) buffer, detergent-containing buffer (TBSX, TBS with 1% Triton X-100), and 70% formic acid (FA) to obtain the buffer-soluble (TBS), detergent-soluble (TBSX) and insoluble (FA) proteins, respectively [35]. Briefly, the frozen brain tissues were homogenized in ice-cold TBS buffer containing a protease inhibitor cocktail (Roche) and a phosphatase inhibitor (Roche) by Polytron homogenizer. The supernatant was collected after centrifugation at 100,000 × g for 60 min at 4 °C as TBS fraction. The residual pellet was re-homogenized in TBSX buffer with protease and phosphatase inhibitors, sonicated, incubated at 4 °C for 30 min with end-over-end agitation, and centrifuged as above to obtain supernatant as the TBSX fraction. The pellet was re-suspended in 70% formic acid, sonicated, and incubated for 12 to 16 h at 4 °C. With the same centrifugation as above, the resultant supernatant (FA fraction) was collected and neutralized 20-fold with 1 M Tris-buffer (pH 11).

### Size-exclusion separation of soluble proteins in TBS fraction

TBS-soluble brain lysates were fractionated by size exclusion chromatography (SEC) with AKTA fast protein liquid chromatography (FPLC) (GE Healthcare) and tandem Superose 6, 10/300 GL columns (GE Healthcare) in phosphate buffer containing 50 mM sodium phosphate, pH 7.4, 150 mM NaCl, and 1 mM EDTA at a flow rate of 0.3 ml/minute and 800 μl/fraction. TBS fractions (80 µl per sample) from 3–4 brain samples with the same *APOE* genotype and pathological diagnosis (AD or AD + LB) were pooled and subjected to SEC.

### Quantification of α-SYN, tau, Aβ40, Aβ42 and APOE by ELISA

The brain lysates from TBS, TBSX and FA fractions, as well as the SEC-separated TBS fractions, were used to assess the amounts of α-SYN, tau, Aβ40, Aβ42 and APOE by ELISA. The amount of α-SYN was measured using a commercially available sandwich ELISA using a mouse monoclonal capture antibody (Anaspec) based on the manufacturer’s protocol. All samples and standards were assayed in duplicates and then averaged. Linearity of dilution/spike-recovery experiments was performed in order to evaluate matrix effects through the addition of known amounts of the manufacturer-provided recombinant α-SYN protein to diluted brain lysates. A spike-recovery range of 99–111% was observed. The levels of tau, Aβ, and APOE in TBS, TBSX and FA fractions in the same AD cohort have been reported previously [35]. The measurements of tau, Aβ and APOE in SEC-separated fractions were performed using the ELISA protocols described previously [35]. Briefly, the levels of tau were determined by ELISA using a monoclonal tau antibody (HT7; Thermo Scientific) as a capture antibody and a biotin-conjugated tau antibody (BT2; Thermo scientific) as a detection antibody. Aβ40 and Aβ42 levels were measured using monoclonal antibodies (13.1.1 for Aβ40 and 2.1.3 for Aβ42) and an HRP-conjugated detection antibody (Ab5). All Aβ antibodies were produced in-house [30]. For APOE ELISA, WUE-4 capture antibody (Novus) and biotin-conjugated detection antibody (K74180B, Meridian Life Science) were used.

### Western blotting

The proteins were resolved by sodium dodecyl sulfate–polyacrylamide gel electrophoresis (SDS-PAGE), and transferred to polyvinylidene difluoride membranes, which were subsequently blocked using 5% milk in PBS. After blocking, proteins were detected with a primary antibody overnight at 4 °C. The next day, membranes were washed, and probed with horseradish peroxide (HRP)-conjugated secondary antibody and developed with enhanced chemiluminescence imaging. The primary antibodies were as follows: anti-α-SYN (BioLegend, 1:1000; BD Biosciences, 1:1000), anti-APOE (K74180B, Meridian Life Science, 1:1000), anti-Tau (HT7, Thermo scientific, 1:500), anti-β-actin (Sigma-Aldrich, 1:2000).

### Detection of α-SYN seeding activity by real-time quaking-induced conversion (RT-QuIC) assay

To investigate the α-SYN seeding activities, we performed RT-QuIC assay with TBS brain lysates from 90 AD samples by randomly selecting 21 and 24 *APOE4*^+^ samples from AD and AD + LB group, respectively, and then selecting the age- and sex-matched *APOE4*^*−*^ cases from the same disease group (Supplementary Table 2, online resource). The TBS brain lysates from 16 normal and 18 LBD brains were also included. RT-QuIC assay was performed as previously described in a 96-well clear-bottom plate with minor modifications (Nalgene Nunc International) [40, 41]. The reaction mixture consisted of final concentrations of 40 mM of phosphate buffer (pH 8.0), 170 mM of NaCl, 10 μM of thioflavin T (ThT), 0.0006% sodium dodecyl sulfate (SDS), and 0.1 mg/ml of recombinant α-SYN (Proteos). To seed the RT-QuIC reactions, 5 μl of the TBS brain lysates were mixed with 95 μl of α-SYN RT-QuIC reaction mixture per well of a 96-well plate preloaded with six 0.8-mm silica beads (OPS Diagnostics). Next, plates were sealed with a plate sealer (Nalgene Nunc International), and reactions were initiated in a FLUOstar Omega plate reader (BMG LABTECH Inc.) with alternating 1-min shake and rest cycles (double orbital, 400 rpm) at 40 °C. ThT fluorescence readings were recorded at excitation and emission wavelengths of 450 and 480 nm, respectively, every 30 min over a period of 60 h. Samples were run in at least triplicates for brain tissues and in duplicates for SEC fractions. Threshold fluorescence was calculated as the average fluorescence of the first 10 cycles for all samples plus 25 standard deviations (SD). The protein aggregation rate (PAR) for each sample was calculated by taking the inverse of the time required to cross the threshold fluorescence calculated in hours. The maximum ThT florescence signal was calculated based on the average of the ThT florescence measured across the last 10 cycles of the reaction plateau for each sample. All the samples were tested in a blinded study design. The experimenter was blinded to the samples after randomization and anonymization prior to testing.

### Measurement of protein concentration in the aggregated product after amplification

The aggregated end products of the RT-QuIC reaction were centrifuged at 20,000*g* for 30 min at 4 °C. After discarding the supernatants, pellets were suspended with PBS. The amount of aggregated product was measured by silver staining after SDS-PAGE. For SDS-PAGE, resuspended pellets were separated on a 12% Bis–Tris gel and protein bands were visualized by silver staining according to the manufacturer’s instructions (Thermo Fisher Scientific).

### Protease K resistance test

Samples containing 3.5 μM α-SYN aggregates amplified by RT-QuIC were treated with 2.5 μg/ml protease K. After 30 min of incubation at 37 °C, the reaction was stopped by heating the sample in SDS sample buffer at 95 °C for 5 min. The input and the digested products were resolved by 12% Bis–Tris gels and the α-SYN was detected by Western blotting as described above.

### Fluorescence resonance energy transfer (FRET) assay

The α-SYN biosensor cells, HEK293T cells transduced with α-SYN (A53T)-CFP and α-SYN (A53T)-YFP lentiviral constructs, were maintained and the FRET assay was conducted as previously described [17, 24, 64]. Briefly, the biosensor cells were plated in 8-well chamber (ibidi) in Opti-MEM (Gibco life technologies) with 10% FBS (HyClone) and grown overnight. The following day, the cells were treated with 0.35 μM amplified α-SYN aggregates or α-SYN monomer in Opti-MEM with 1 μl of Lipofectamine 2000 (Invitrogen) to a total volume of 20 μl/well. After 72 h of incubation, cells were fixed in 4% paraformaldehyde for 10 min, then subjected to FRET assay. FRET signals were captured using excitation at 405 nm and detection range at 530–650 nm by a confocal microscope. To quantify α-SYN inclusions in FRET channel, images were processed and quantified with custom scripts written in Matlab (r2020b) to detect inclusions, using the same parameter settings across all images to allow unbiased quantification. Briefly, images were first thresholded to create binary images. Next, images were morphologically closed with Matlab’s “imclose”, using a square structural element of width 10 pixels. Regions of interest were detected with Matlab’s “regionprops”, and only those regions with an eccentricity less than 0.8 were kept. Finally, regions were filtered to have areas between 4 and 1000 pixels using Matlab’s “bwareafilt”. The intensities of inclusions were quantified by summing the raw pixel intensities of each region.

### Culture and differentiation of iPSC-derived neurons

Human iPSCs were differentiated into neurons as previously described [10, 67]. Briefly, iPSCs were cultured in commercial neural induction medium (Stemcell Technologies) according to the manufacturer’s instructions with some modifications. To initiate neurosphere formation, iPSC clumps were cultured in a neural induction medium in suspension for 5–7 days in 6-well plates. To induce neural rosette formation, the neurospheres were seeded onto matrigel-coated dishes and cultured in a neural induction medium for another 5–7 days. Neural rosettes were isolated as a single cell suspension and re-plated onto matrigel-coated dishes in a neural induction medium. To differentiate into neural progenitor cells (NPCs), the medium was replaced with a neural progenitor cell medium (Stemcell Technologies) and cultured for additional 10–14 days. NPCs were amplified and frozen stocks were made for further experiments. For neuronal differentiation, NPCs were seeded on matrigel-coated plates in a neural progenitor cell medium. The following day, the media was replaced to neuronal differentiation medium to differentiate NPCs into neurons for additional 14 days, which was composed of DMEM/F12 and Neurobasal Medium (1:1) supplemented with N2, B27, BDNF (20 ng/ml), GDNF (20 ng/ml), NT3 (10 ng/ml), IGF (10 ng/ml), ascorbic acid (200 μM) (all from Stemcell Technologies) and dbcAMP (100 nM) (Sigma Aldrich).

### Immunofluorescence staining for characterizing iPSC-derived neurons

The iPSC-derived neurons at DIV 14 were fixed in 4% PFA, permeabilized in 0.2% Triton X-100 and blocked in 2% bovine serum albumin for 1 h and incubated overnight with the primary antibodies of TUJ1 (β-III tubulin, Sigma-Aldrich, 1:500), MAP2 (Microtubule-associated protein 2, Abcam, 1:1000), vGLUT1 (Vesicular glutamate transporter 1, Synaptic systems, 1:100), GAD67 (Glutamic acid decarboxylase 67, Merck Millipore, 1:100), and TH (Tyrosine hydroxylase, Merck Millipore, 1:100). Cells were then incubated with Alexa Fluor-conjugated secondary antibodies for 2 h at room temperature (1:400, Invitrogen). After DAPI staining, images were scanned using a confocal microscope.

### Neurite length measurement

The iPSC-derived neurons at DIV 12 were treated with 0.35 μM amplified α-SYN aggregates. After 48 h of incubation, cells were fixed in 4% PFA, permeabilized in 0.2% Triton X-100 and blocked in 2% bovine serum albumin for 1 h and incubated overnight with the primary antibody of TUJ1 (Sigma-Aldrich, 1:500). Cells were then incubated with Alexa Fluor-conjugated secondary antibody for 1 h at room temperature (1:1000, Invitrogen). Images were scanned using a confocal microscope by taking 2–3 images from 3 independent samples per experimental group. Total 340 to 480 neurons per experimental group were analyzed to quantify the neurite length.

Image quantification was performed with custom scripts written in MATLAB (r2020b). Briefly, 8-bit images were pre-processed with a nonlinear filter over red (TUJ1) and blue (DAPI) channels by first rescaling images onto [0,1], linearly transforming (parameterized by y = m(x – b) + 0.5), then applying the tanh function, and finally re-normalizing values to fall within 0–255. For red channels, values of m = 2 and b = 0.4 were used. Across all images, the same parameter settings were used. Nuclei and neurite were detected by eroding images with a square structural element and detecting connected regions above a threshold value and of sufficient area. Detected nuclei were dilated, then subtracted from the neurite channel, before detecting neurites. Neurite length per nuclei was computed by summing the total neurite length in an image, then dividing by the total number of nuclei after skeletonizing neurites.

### MTT assay

Human iPSC-derived neurons at DIV 12 were treated with 0.35 μM amplified α-SYN aggregates or α-SYN monomer. After 48 h of incubation, cell viability of the neurons was assessed by 3-(4,5-dimethylthiazol-2-yl)-2,5-diphenyl-2H-tetrazolium bromide (MTT) assay according to the manufacturer’s instructions (Sigma-Aldrich). MTT labeling reagent was added to each well and incubated for 4 h, followed by addition of solubilization solution into each well. Wavelengths between 550 and 600 nm were measured for analyzing cell viability of neurons.

### Statistical analysis – functional studies and the cohort of control and LBD brains

All data were reported as mean values ± SEM unless elsewise indicated. To ensure that results were valid in the presence of non-normal distributions, or differing variances between groups, nonparametric Mann–Whitney U tests between-group comparisons with Bonferroni correction for multiple comparisons, and Kruskal–Wallis tests with Dunn’s multiple comparison tests were used to compare outcomes when sample size are larger than 8. With the sample size ≤ 8, since nonparametric tests would have very low power, a student *t* test or one-way ANOVA was used to compare outcomes among groups. Mean values for multiple groups were compared using two-way ANOVA comparison tests. Statistical analyses were performed using GraphPad Prism v8.4.3 (GraphPad Software).

### Statistical analysis – AD cohort

Specifically, continuous variables were summarized using the sample median (minimum, maximum). Categorical variables were summarized with number and percentage. Associations of tau, Aβ40, Aβ42 and APOE (all in TBS, TBSX and FA fractions) with the three separate outcomes of α-SYN TBS, α-SYN TBSX, and α-SYN FA were evaluated using single-variable (i.e. unadjusted) and multivariable linear regression models in AD and AD + LB groups. Multivariable models were adjusted for age at death, sex, CAA score, number of *APOE4* alleles, Braak neurofibrillary tangle stage and Thal amyloid phase. Predictor variables and α-SYN outcomes were transformed as needed for use in linear regression analysis to address distributional skewness (Supplementary Table 3, online resource). Regression coefficients and 95% confidence intervals (CIs) were estimated and are interpreted as the change in mean outcome level corresponding to the presence of the given characteristic (categorical measures) or corresponding to each 1-unit increase in the given measure (on the untransformed, square-root transformed, or natural logarithm transformed scale, Supplementary Table 3, online resource).

Associations of tau, Aβ40, Aβ42, α-SYN and APOE (again, all in TBS, TBSX and FA fractions) with two different RT-QuIC signals [protein aggregation rate (PAR) and maximum fluorescence (MaxFL)] were assessed with proportional odds logistic regression models, where the RT-QuIC outcomes were converted into ordinal variables due to their non-normal distributions. Specifically, PAR was converted to a three-level categorical variable based on the 50% and 75% percentiles, and MaxFL was converted to a four-level categorical variable based on the 25%, 50% and 75% percentiles (Supplementary Table 3, online resource). Models were adjusted for age at death, sex, CAA score, Braak neurofibrillary tangle stage and Thal amyloid phase, with and without further adjustment of the number of *APOE4* alleles. Predictor variables were transformed due to distributional skewness as previously described. Odds ratios (ORs) and 95% CIs were estimated and are interpreted as a multiplicate increase in the odds being in a higher RT-QuIC outcome category. Comparisons of α-SYN TBS, α-SYN TBSX, α-SYN FA, PAR, and MaxFL between AD and AD + LB groups, and according to the presence of the *APOE4* allele in the separate AD and AD + LB groups, we used linear and proportional odds logistic regression models as previously described.

To adjust for multiple testing, we utilized a Bonferroni correction separately for each family of similar statistical tests (see table footnotes or figure legends for details). All statistical tests were two-sided. Statistical analyses were performed using R Statistical Software (version 3.6.1; R Foundation for Statistical Computing).

## Results

### Increased α-SYN aggregates in AD brains with LB pathology

**Fig. 1:**
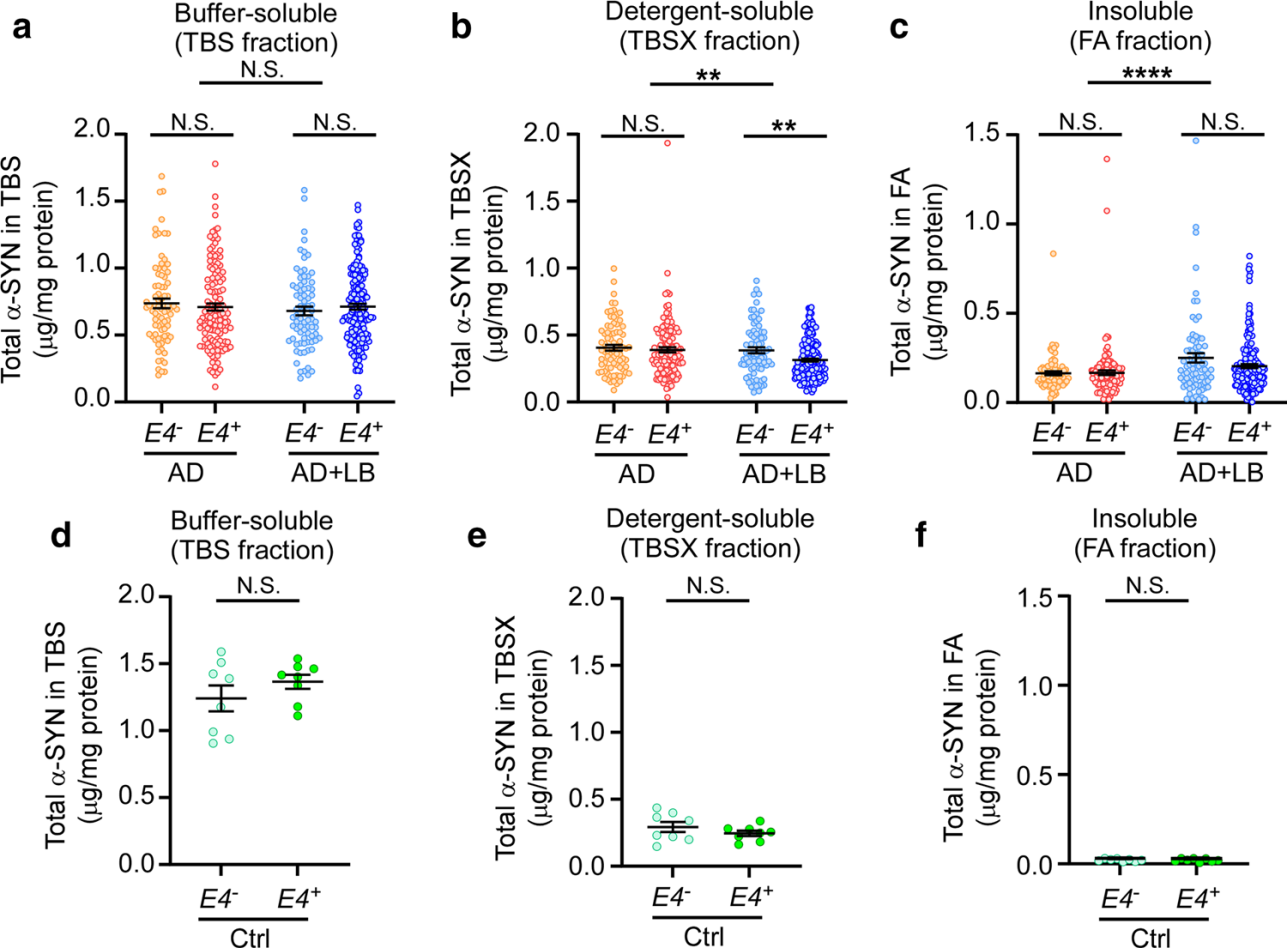
Levels of α-SYN in the brain samples from patients with AD and controls. The brain samples from the superior temporal lobe of patients with AD (with or without LB co-pathologies) **a**-**c** and controls (Ctrl) **d**-**f** were lysed sequentially in TBS (buffer-soluble fraction), TBSX (TBS with 1% Triton X-100, detergent-soluble fraction), and FA (70% formic acid, insoluble fraction) buffer. The levels of total α-SYN in the TBS (**a** and **d**), TBSX (**b** and **e**), and FA (**c** and **f**) fractions were measured by ELISA and compared between AD and AD + LB patients with or without *APOE4* gene (*E4*^*−*^ and *E4*^+^) **a–c**, or controls with or without *APOE4* gene **d–f**. Data represent mean ± SEM. *N* = 469 samples in total for AD and AD with LB co-pathologies. Comparisons of α-SYN between *E4*^*−*^ and *E4*^+^ subjects, and between AD and AD + LB subjects, were made using linear regression models that were adjusted for age at death, sex, CAA score, Braak stage, Thal phase, and number of *APOE4* alleles (only for AD vs. AD + LB comparisons), where each α-SYN outcome was examined on the square root scale. *N* = 16 samples in total for control cases. Mann–Whitney *U* tests were used for statistical analyses. **p < 0.01; ****p < 0.0001; *N.S*. not significant

To evaluate α-SYN aggregates and assess the potential effects of *APOE4* in the presence of Aβ, we used autopsy-confirmed AD cases (N = 469) (Supplementary Table 1, online resource) [35] with 214 (46%) cases having no α-SYN pathology (AD) and 255 cases (54%) having α-SYN pathology (AD + LB). To explore the effects of *APOE4* on α-SYN, we further subdivided the cohort into *APOE4* non-carriers (*APOE4*^*−*^, including *APOE2/3* and *APOE3/3*, *N* = 80 in AD group and *N* = 78 in AD + LB group) and *APOE4* carriers (*APOE4*^+^, including *APOE2/4*, *APOE3/4* and *APOE4/4*, N = 134 in AD group and *N* = 177 in AD + LB group) (Table 1). We quantified the amounts of total α-SYN in the brain lysates of the superior temporal gyrus after sequentially extracting proteins in TBS (buffer-soluble), TBSX (detergent-soluble), and FA (insoluble) fractions. We did not detect any significant differences of total α-SYN in the buffer-soluble (TBS) fractions between AD and AD + LB, with or without *APOE4* gene allele (Fig. 1a). Interestingly, the amount of α-SYN was significantly decreased in detergent-soluble (TBSX) fraction in AD + LB compared to AD. This fraction likely contains membrane-bound proteins, and the decrease was most prominent in AD + LB cases carrying an *APOE4* allele (Fig. 1b). Additionally, we found that AD + LB had significantly higher α-SYN levels in insoluble (FA) fractions compared with AD, as expected (Fig. 1c). The levels of α-SYN in insoluble fractions did not differ between *APOE4* non-carriers and carriers (Fig. 1c). Furthermore, we evaluated α-SYN levels in a relatively small cohort of autopsy-confirmed control brains (*N* = 16, 8 age- and sex-matched samples/*APOE* genotype) (Table 2). We found that the α-SYN levels in the insoluble FA fractions were very low in these control brains as expected, and we did not find a significant difference in the levels of α-SYN in TBS, TBSX, or FA fractions between *APOE4* non-carriers and carriers (Fig. 1d-f). Altogether, these results suggest that the distribution of α-SYN shifted from soluble to insoluble fractions in AD cases, in particular, AD brains with LB pathology; and that *APOE4* gene did not significantly affect the amount of insoluble α-SYN, although it was associated with less detergent-soluble α-SYN in AD + LB brains.

### α-SYN aggregates correlate with AD-related pathologies

Next, we evaluated whether the amount of total α-SYN correlated with the other hallmarks of AD. We performed correlation analyses between levels of α-SYN and those of total tau, Aβ40, Aβ42 and APOE measured in the same brain lysates using multivariable analyses [35]. In the AD brains without LB, we observed a significant (*p* < 0.0042 considered significant after multiple testing corrections) association between the levels of α-SYN in TBS with Aβ42 in TBSX fractions, α-SYN in TBSX with Tau in TBSX and FA fractions, α-SYN in TBSX with Aβ42 in all three fractions, as well as α-SYN in FA with tau in FA fractions, after adjusting for age at death, sex, CAA score, number of *APOE4* alleles, Braak stage, and Thal phase (Table 3). In the AD + LB brains, the correlations mentioned above were preserved with more pronounced associations (Table 4). In particular, we found a significant correlation between the insoluble α-SYN and insoluble Aβ40, Aβ42, and APOE which were not observed in AD brains without LB (Table 4; Supplementary Fig. 1a–d, online resource), suggesting that the presence of LB may intensify associations between α-SYN aggregates and AD-related pathologies in this brain region.

**Table 3:** Associations of variables with α-SYN in the subgroup of 214 AD patients without LB pathology

Variable	Association with α-SYN in TBS		Association with α-SYN in TBSX		Association with α-SYN in FA	
	Regression coefficient (95% CI)	*P*-value	Regression coefficient (95% CI)	*P*-value	Regression coefficient (95% CI)	*P*-value
Total tau TBS	1.25 (0.33, 2.17)	0.008	– 0.56 ( – 1.34, 0.23)	0.16	– 0.20 ( – 0.81, 0.40)	0.51
Total tau TBSX	0.10 (0.03, 0.17)	0.005	0.16 (0.11, 0.21)	** < 0.001**	0.03 ( – 0.01, 0.08)	0.17
Total tau FA	0.23 ( – 0.56, 1.02)	0.57	1.82 (1.20, 2.43)	** < 0.001**	1.44 (0.95, 1.94)	** < 0.001**
Aβ40 TBS	– 0.28 ( – 0.80, 0.24)	0.28	0.01 ( – 0.43, 0.45)	0.97	– 0.07 ( – 0.41, 0.27)	0.68
Aβ40 TBSX	– 0.17 ( – 1.36, 1.01)	0.77	– 0.87 ( – 1.90, 0.15)	0.094	– 0.06 ( – 0.86, 0.75)	0.88
Aβ40 FA	– 0.13 ( – 0.72, 0.46)	0.66	0.41 ( – 0.08, 0.90)	0.10	0.31 ( – 0.07, 0.69)	0.11
Aβ42 TBS	1.09 ( – 0.14, 2.32)	0.083	2.65 (1.67, 3.62)	** < 0.001**	0.65 ( – 0.15, 1.46)	0.11
Aβ42 TBSX	3.37 (1.71, 5.03)	** < 0.001**	3.86 (2.51, 5.22)	** < 0.001**	0.79 ( – 0.34, 1.92)	0.17
Aβ42 FA	1.60 (0.48, 2.72)	0.005	2.75 (1.87, 3.63)	** < 0.001**	1.07 (0.31, 1.83)	0.006
APOE TBS	0.16 ( – 0.01, 0.32)	0.068	0.11 ( – 0.03, 0.25)	0.14	0.02 ( – 0.09, 0.14)	0.67
APOE TBSX	0.34 (0.10, 0.57)	0.005	– 0.18 ( – 0.38, 0.02)	0.072	– 0.21 ( – 0.36, − 0.06)	0.008
APOE FA	0.19 ( – 0.85, 1.22)	0.72	– 0.01 ( – 0.88, 0.86)	0.99	– 0.04 ( – 0.74, 0.66)	0.91

*CI*  confidence interval. Regression coefficients, 95% CIs, and p-values result from linear regression models that were adjusted for age at death, sex, CAA score, number of *APOE4* alleles, Braak stage, and Thal phase, and where α-SYN TBS, α-SYN TBSX and α-SYN FA were considered on the square root scale. Regression coefficients are interpreted as the change in the mean outcome level (α-SYN TBS or α-SYN TBSX or α-SYN FA, all on the square root scale) corresponding to each 1-unit increase in the given variable (on the untransformed, square root, or natural logarithm transformed). *P*-values ≤ 0.0042 were considered as statistically significant after applying a Bonferroni correction for multiple testing separately for each α-SYN measure; statistically significant associations are shown in bold

**Table 4:** Associations of variables with α-SYN in the subgroup of 255 AD patients with LB pathologies

Variable	Association with α-SYN in TBS		Association with α-SYN in TBSX		Association with α-SYN in FA	
	Regression coefficient (95% CI)	*P*-value	Regression coefficient (95% CI)	*P*-value	Regression coefficient (95% CI)	*P*-value
Total tau TBS	2.36 (1.40, 3.32)	** < 0.001**	0.53 ( – 0.26, 1.32)	0.19	– 0.57 ( – 1.51, 0.37)	0.23
Total tau TBSX	0.14 (0.07, 0.21)	** < 0.001**	0.23 (0.18, 0.27)	** < 0.001**	– 0.05 ( – 0.11, 0.02)	0.17
Total tau FA	1.23 (0.42, 2.05)	0.003	1.85 (1.24, 2.45)	** < 0.001**	2.45 (1.74, 3.16)	** < 0.001**
Aβ40 TBS	– 0.69 ( – 1.14, – 0.23)	0.003	0.11 ( – 0.26, 0.48)	0.55	– 0.04 ( – 0.47, 0.40)	0.86
Aβ40 TBSX	– 0.16 ( – 1.04, 0.71)	0.71	– 0.70 ( – 1.38, – 0.01)	0.047	0.12 ( – 0.70, 0.94)	0.78
Aβ40 FA	– 0.17 ( – 0.65, 0.31)	0.49	0.27 ( – 0.11, 0.65)	0.16	0.84 (0.40, 1.28)	** < 0.001**
Aβ42 TBS	1.88 (0.64, 3.12)	0.003	2.95 (2.07, 3.82)	** < 0.001**	0.94 ( – 0.18, 2.06)	0.10
Aβ42 TBSX	3.89 (2.59, 5.18)	** < 0.001**	3.45 (2.49, 4.40)	** < 0.001**	0.88 ( – 0.36, 2.12)	0.17
Aβ42 FA	2.10 (1.17, 3.03)	** < 0.001**	2.33 (1.64, 3.03)	** < 0.001**	1.43 (0.55, 2.30)	**0.002**
APOE TBS	0.25 (0.10, 0.40)	**0.001**	0.06 ( – 0.06, 0.18)	0.33	– 0.05 ( – 0.19, 0.09)	0.48
APOE TBSX	0.19 (-0.04, 0.43)	0.10	– 0.46 ( – 0.63, – 0.28)	** < 0.001**	– 0.04 ( – 0.26, 0.18)	0.73
APOE FA	0.50 (-0.42, 1.42)	0.29	0.31 ( – 0.42, 1.04)	0.40	1.70 (0.86, 2.54)	** < 0.001**

*CI *confidence interval. Regression coefficients, 95% CIs, and *p*-values result from linear regression models that were adjusted for age at death, sex, CAA score, number of *APOE4* alleles, Braak stage, and Thal phase, and where α-SYN TBS, α-SYN TBSX and α-SYN FA were considered on the square root scale. Regression coefficients are interpreted as the change in the mean outcome level (α-SYN TBS or α-SYN TBSX or α-SYN FA, all on the square root scale) corresponding to each 1-unit increase in the given variable (on the untransformed, square root, or natural logarithm transformed). *P*-values ≤ 0.0042 were considered as statistically significant after applying a Bonferroni correction for multiple testing separately for each α-SYN measure; statistically significant associations are shown in bold

### APOE4 exacerbates α-SYN seeding activity

**Fig. 2:**
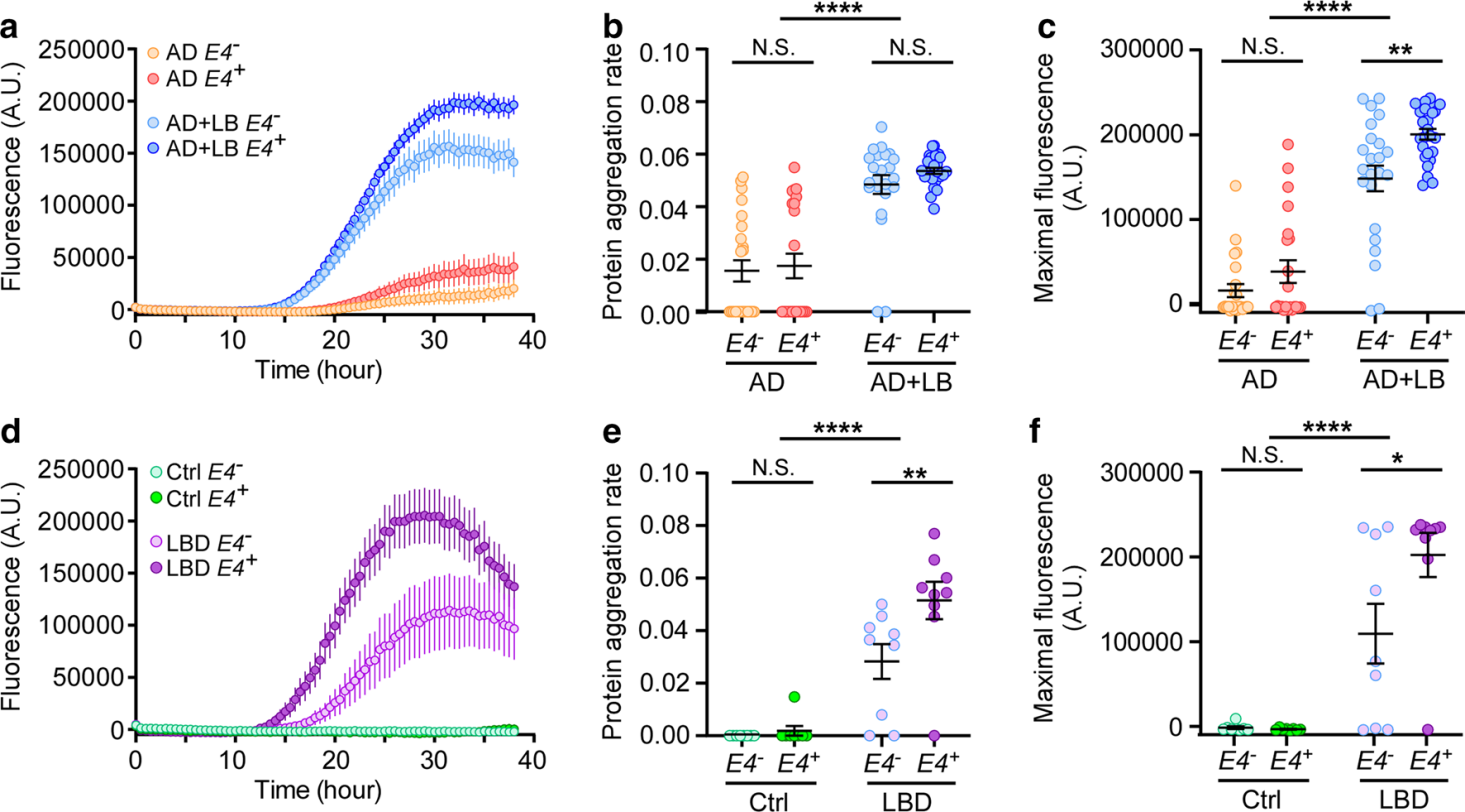
Effects of LB pathology and *APOE4* gene allele on α-SYN seeding activities. Samples of TBS brain lysate from patients with AD, AD + LB, LBD, or controls, with or without *APOE4* gene allele, were subjected to α-SYN RT-QuIC assay. **a** and **d** The extent of aggregation was monitored by ThT fluorescence and the aggregation curve is shown. The protein aggregation rate (PAR, **b** and **e**) and the maximum fluorescence values (A.U., measured at plateau of aggregation, c and f) are shown. Each dot represents the average value of an individual biological sample measured in triplicate. Data are mean ± SEM. *N* = 21–24 samples/group for AD and AD + LB. Comparisons of RT-QuIC outcomes between *E4*^*−*^ and *E4*^+^ subjects, and between AD and AD + LB subjects, were made using proportional odds logistic regression models that were adjusted for age at death, sex, CAA score, Braak stage, Thal phase, and number of *APOE4* alleles (only for AD vs. AD + LB comparisons), where each α-SYN outcome was examined as an ordered categorical variable. *N* = 8 samples/*APOE* genotype in controls and 9 samples/*APOE* genotype in LBD. Two-way ANOVA with Sidak’s multiple comparison tests were used for statistical analyses. *p < 0.05, **p < 0.01, ****p < 0.0001, *N.S.* not significant

The misfolding and aggregation of α-SYN involves a mechanism of seeding and nucleation in which initial seeds of α-SYN recruit other soluble monomers that assemble to form aggregates [63]. The α-SYN RT-QuIC assay uses the seeding–nucleation mechanism to cyclically amplify the process of protein misfolding, enabling the efficient amplification of small quantities of α-SYN oligomers and thereby facilitating their detection [20, 42]. To investigate whether *APOE4* can affect the α-SYN seeding activities, we performed RT-QuIC assay in 90 samples of TBS brain lysates from AD and from AD + LB that were matched for age and sex, with or without *APOE4*, using a blinded study design as described in methods (Supplementary Table 2, online resource). As expected, AD + LB had a significantly higher RT-QuIC signal compared with AD, reflected by significantly higher PAR (Fig. 2a and b) and maximum ThT signal (Fig. 2a and c). Of note, 2 of the 47 samples from AD + LB of *APOE4* non-carriers had negative RT-QuIC response (Fig. 2b and c). Conversely, 19 of 43 samples (44%) from AD showed positive RT-QuIC response with varying degrees of aggregation profile (Fig. 2a–c), implying that potential α-SYN aggregates were present even though no LB were detected in neuropathologic evaluations. Importantly, in AD + LB, the maximum ThT fluorescence was significantly greater in samples from *APOE4* carriers than in *APOE4* non-carriers (Fig. 2c); suggesting that APOE4 might play a role in exacerbating α-SYN seeding activity in AD brains.

To further evaluate the role of APOE4 in α-SYN seeding activity, we performed RT-QuIC assays with a relatively small cohort of autopsy-confirmed LBD brains with minimal Alzheimer type pathology (*N* = 18, 9 age- and sex-matched samples/*APOE* genotype) and control brains (*N* = 16, 8 age- and sex-matched samples/*APOE* genotype) (Table 2). As expected, we observed a positive RT-QuIC signal in LBD cases but not from control cases, reflected by significantly higher PAR and maximum ThT signal (Fig. 2d–f). Interestingly, in these LBD cases without AD pathology, *APOE4* carriers showed significantly higher PAR and maximum ThT signal than *APOE4* non-carriers (Fig. 2e and f). Altogether, these results indicate that *APOE4* exacerbates α-SYN seeding activity independent to Aβ.

To confirm the reproducibility of our RT-QuIC assay, we calculated the intra-batch and inter-batch percentage of coefficient of variations (CV%) using samples from control and LBD brains as negative and positive samples, respectively. There were no significant differences between intra-batch and batch-to-batch runs in the PAR and maximum ThT signals (Supplementary Fig. 2a–f, online resource). Similar to a previous report [52], the negative control samples showed higher CV% of maximum ThT signals compared to the positive control samples due to the very low values. The CV% for these two parameters are provided in Supplementary Fig. 2a–f (online resource).

### The seeding activity of α-SYN is associated with the amounts of α-SYN, APOE and Aβ40

Next, we examined what AD-related markers might affect the α-SYN seeding activity. We analyzed associations between protein aggregation rate (PAR), maximum ThT signals by RT-QuIC and the levels of α-SYN, tau, Aβ40, Aβ42 and APOE in the TBS, TBSX and FA fractions (Tables 5 and 6). After correcting for multiple testing (*p* < 0.0031 considered significant), the presence of LB was significantly associated with both PAR (OR = 38, *p* < 0.001) (Table 5) and maximum RT-QuIC ThT signals (OR = 45, *p* < 0.001) (Table 6) adjusting for age, sex, CAA score, Braak stage, Thal phase, and *APOE4* genotype. The level of insoluble α-SYN in FA fractions was significantly correlated with PAR (OR = 1.14, p < 0.001), suggesting that the amount of α-SYN aggregates influences the initiation of soluble α-SYN seeding activity (Table 5). More interestingly, we found the amount of insoluble APOE in FA fractions was strongly correlated with the maximum RT-QuIC ThT signals (OR = 1.09, *p* < 0.001), with a similar, though slightly weaker, nominally significant (*p* < 0.05) association observed when adjusting for a number of *APOE4* alleles (OR = 1.07, *p* = 0.01, Table 6). Given that the ThT signal reflects the amount of β-sheet-rich structures of α-SYN, our finding implies that the presence of APOE, in particular APOE4, may further modify α-SYN conformation thus leading to higher seeding activity. We did not find any correlation between PAR and maximum RT-QuIC ThT signals with levels of tau and Aβ42 in any of the fractions (Table 5 and 6). Levels of Aβ40 in TBS and FA fractions were significantly correlated with maximum RT-QuIC ThT signals before adjusting for *APOE4* gene allele (TBS: OR = 1.44, *p* < 0.001; FA: OR = 1.50, *p* < 0.001), and nominally significant after adjusting for *APOE4* gene allele (TBS: OR = 1.41, *p* = 0.01; FA: OR = 1.39, *p* = 0.03). Altogether, our findings suggest that α-SYN, APOE, and Aβ40 influence α-SYN seeding ability and that Aβ42 and tau may play a less important role in α-SYN seeding in AD.

**Table 5:** Associations of variables with RT-QuIC protein aggregation rate (PAR)

Variable	Adjusting for age at death, sex, CAA score, Braak stage, and Thal phase		Additional adjustment for the number of *APOE4* alleles	
	OR (95% CI)	*P*-value	OR (95% CI)	*P*-value
LB presence	**34.53 (11.26, 125.12)**	** < 0.001**	**37.85 (11.85, 144.45)**	** < 0.001**
Total tau TBS	1.83 (0.92, 3.88)	0.086	1.97 (0.98, 4.23)	0.059
Total tau TBSX	0.99 (0.93, 1.04)	0.62	0.99 (0.93, 1.05)	0.67
Total tau FA	1.29 (0.86, 1.97)	0.22	1.26 (0.84, 1.93)	0.27
Aβ40 TBS	1.26 (0.97, 1.67)	0.088	1.26 (0.96, 1.66)	0.10
Aβ40 TBSX	1.46 (0.82, 2.61)	0.20	1.45 (0.81, 2.59)	0.20
Aβ40 FA	1.24 (0.93, 1.66)	0.15	1.20 (0.89, 1.63)	0.22
Aβ42 TBS	1.72 (0.69, 4.38)	0.24	1.94 (0.77, 5.05)	0.16
Aβ42 TBSX	1.59 (0.68, 3.86)	0.29	1.58 (0.67, 3.86)	0.30
Aβ42 FA	1.20 (0.78, 1.87)	0.40	1.24 (0.80, 1.96)	0.33
α-SYN TBS	0.99 (0.9, 1.08)	0.77	0.98 (0.90, 1.08)	0.73
α-SYN TBSX	0.92 (0.81, 1.04)	0.17	0.91 (0.80, 1.03)	0.14
α-SYN FA	**1.14 (1.05, 1.26)**	** < 0.001**	**1.14 (1.05, 1.25)**	** < 0.001**
APOE TBS	1.01 (0.93, 1.10)	0.82	1.03 (0.94, 1.13)	0.48
APOE TBSX	1.11 (0.96, 1.29)	0.17	1.20 (1.01, 1.42)	0.038
APOE FA	1.03 (0.99, 1.08)	0.16	1.03 (0.98, 1.08)	0.25

*CI* confidence interval. OR = odds ratio. ORs, 95% CIs, and *p*-values result from proportional odds logistic regression models where PAR was categorized into the low threshold, median threshold and high threshold based on the 50% and 75% quantiles of the data. For LB presence, ORs are interpreted as the risk of having a higher order or lower order of seeding activity corresponding to LB presence. For continuous variables, ORs are interpreted as the risk change in having a higher order or lower order of seeding activity corresponding to each 1-unit increase in the given variable (on the untransformed, square root, or natural logarithm transformed). *P*-values ≤ 0.0031 were considered statistically significant after applying a Bonferroni correction for multiple testing; statistically significant associations are shown in bold

**Table 6:** Associations of variables with RT-QuIC maximum fluorescence signals

Variable	Adjusting for age at death, sex, CAA score, Braak stage, and Thal phase		Additional Adjustment for the number of *APOE4* alleles	
	OR (95% CI)	*P*-value	OR (95% CI)	*P*-value
LB presence	**50.14 (16.15, 184.51)**	** < 0.001**	**45.35 (14.46, 168.2)**	** < 0.001**
Total tau TBS	1.30 (0.68, 2.51)	0.42	1.49 (0.77, 2.97)	0.23
Total tau TBSX	0.95 (0.90, 1.01)	0.10	0.96 (0.90, 1.02)	0.15
Total tau FA	1.52 (1.02, 2.33)	0.041	1.44 (0.95, 2.20)	0.083
Aβ40 TBS	**1.44 (1.12, 1.89)**	** < 0.001**	1.41 (1.09, 1.84)	0.009
Aβ40 TBSX	1.66 (0.97, 2.96)	0.066	1.60 (0.93, 2.82)	0.089
Aβ40 FA	**1.50 (1.14, 2.01)**	** < 0.001**	1.39 (1.04, 1.88)	0.025
Aβ42 TBS	1.24 (0.50, 3.08)	0.64	1.45 (0.57, 3.69)	0.43
Aβ42 TBSX	1.40 (0.66, 3.04)	0.38	1.39 (0.64, 3.06)	0.40
Aβ42 FA	0.97 (0.66, 1.44)	0.90	1.04 (0.69, 1.56)	0.86
α-SYN TBS	1.03 (0.94, 1.12)	0.54	1.03 (0.94, 1.12)	0.58
α-SYN TBSX	0.89 (0.79, 1.00)	0.042	0.87 (0.77, 0.98)	0.017
α-SYN FA	1.05 (0.99, 1.13)	0.11	1.05 (0.98, 1.12)	0.14
APOE TBS	1.01 (0.93, 1.09)	0.88	1.06 (0.97, 1.16)	0.18
APOE TBSX	1.07 (0.93, 1.23)	0.35	1.23 (1.04, 1.45)	0.013
APOE FA	**1.09 (1.03, 1.15)**	** < 0.001**	1.07 (1.02, 1.14)	0.010

*CI*  confidence interval. *OR*  odds ratio. ORs, 95% CIs, and *p*-values result from proportional odds logistic regression models where the maximum fluorescence was categorized into no seeding activity, low seeding activity, median seeding activity, and high seeding activity based on the 25%, 50%, and 75% quantile of the data. For categorical variables, ORs are interpreted as the risk of having a higher order or lower order of seeding activity corresponding to the presence of the given characteristic. For continuous variables, ORs are interpreted as the risk change in having a higher order or lower order of seeding activity corresponding to each 1-unit increase in the given variable (on the untransformed, square root, or natural logarithm transformed). *P*-values ≤ 0.0031 were considered statistically significant after applying a Bonferroni correction for multiple testing. The statistically significant results are highlighted in bold

### The presence of LB or *APOE4* impacts the size distribution of APOE and α-SYN proteins

**Fig. 3:**
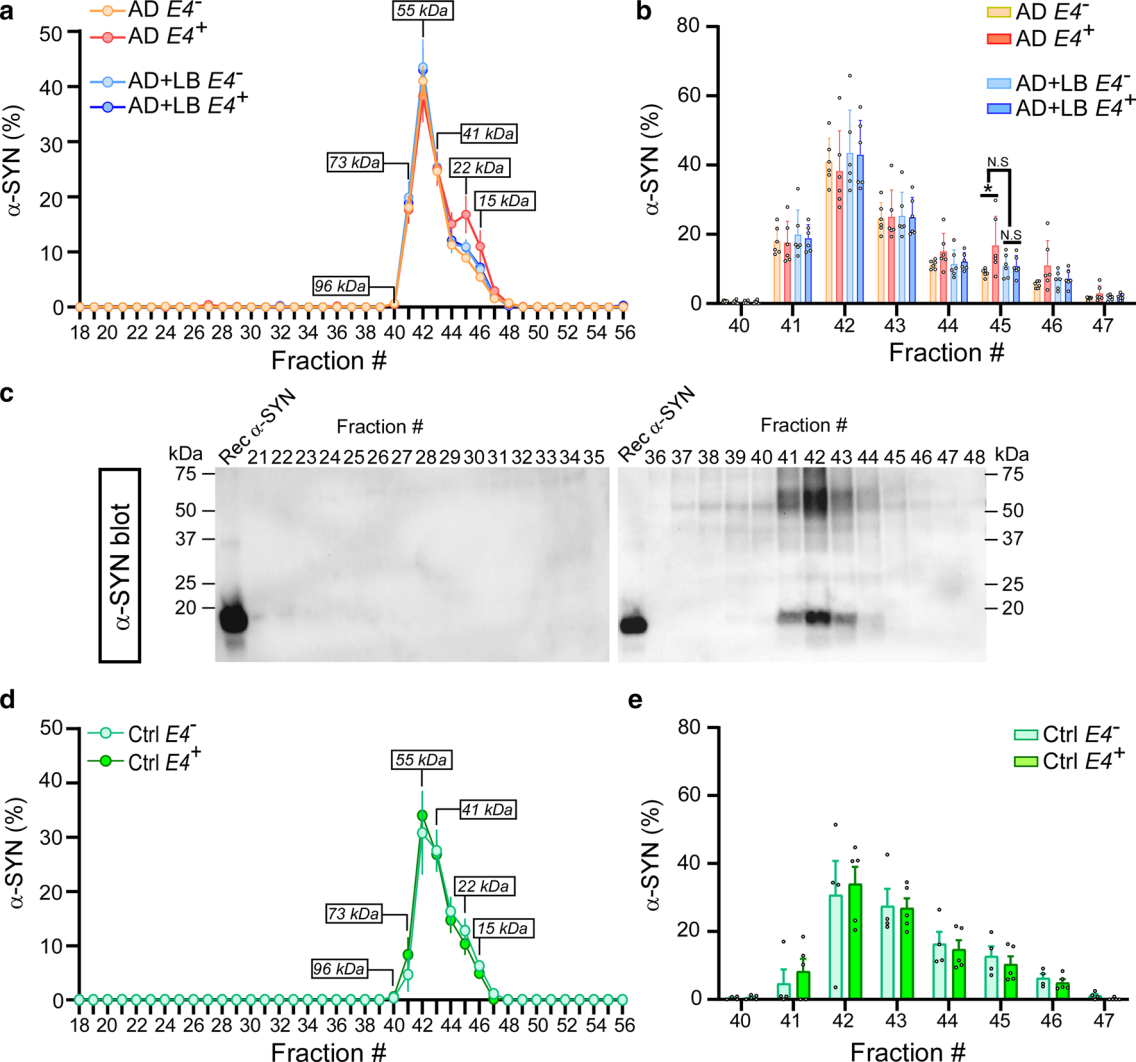
The size distribution of α-SYN in TBS brain lysates from AD and control brains. TBS-soluble human brain lysates from AD, AD + LB, and control brains were fractioned by size exclusion chromatography (SEC) using AKTA FPLC with tandem Superose 6, 10/300 GL columns. The fractions from #18 to #56 were collected and the levels of α-SYN were measured by ELISA and Western blotting. **a** and **b** The size distribution of α-SYN in the AD and AD + LB brains, without or with *APOE4* gene allele, were quantified and compared. Molecular weight markers (kDa) for the peak fractions are indicated. *N* = 6 samples/group. Each sample was mixed with equal amount of TBS lysate from 3–4 brains with the same *APOE* genotype and the same pathological diagnosis (AD or AD + LB). Two-way ANOVA with Sidak’s multiple comparison tests were used for statistical analyses. **c** The distribution of the α-SYN in different fractions was validated using Western blotting with AD samples. **d** and **e** The size distribution of α-SYN in the control brains, without or with *APOE4* gene allele, were quantified and compared. *N* = 4–5 samples/group. The student *t* tests were used for statistical analyses. Data are shown as mean ± SEM. *p < 0.05; *N.S.* not significant. Only the significantly changed fractions were labeled

It has been reported that physiological α-SYN exists as a helically folded tetramer in the brain and that destabilization of the α-SYN tetramer precedes α-SYN misfolding and aggregation in the pathogenesis of synucleinopathies [3]. We evaluated whether the presence of both LB and APOE4 impact the structure of soluble α-SYN in AD. We fractionated TBS brain lysates by size-exclusion chromatography (SEC) using fast protein liquid chromatography (FPLC) with tandem Superose-6, 10/300 GL columns [68]. We then determined the size distribution of α-SYN, tau, Aβ40, Aβ42 and APOE in different fractions by ELISA and Western blotting. We found that the peak of soluble α-SYN (40% of total α-SYN) was ~ 55 kDa in the TBS brain lysates of AD brains (fraction #42, Fig. 3a–c), which is consistent with previous reports by Bartels, et al*.* of α-SYN tetramers [3]. Around 10% of α-SYN was in fractions #45 or #46, which corresponds to the size of α-SYN monomers (Fig. 3a and b). The peak of soluble α-SYN (35% of total α-SYN) around ~ 55 kDa was also observed in control brains, implying that α-SYN size distribution is not affected by AD pathology (Fig. 3d and e). Next, we analyzed the impact of LB pathology and *APOE4* gene allele on the size distribution of α-SYN. We did not observe any difference in α-SYN size distribution in any fractions between AD and AD + LB; however, AD *APOE4* carriers had more monomeric α-SYN in fraction #45 than *APOE4* non-carriers (Fig. 3a and b) which was not shown in the control cases (Fig. 3d and e). This finding might suggest a potential role of APOE4 in regulating the conformational change of α-SYN in AD [3].

**Fig. 4:**
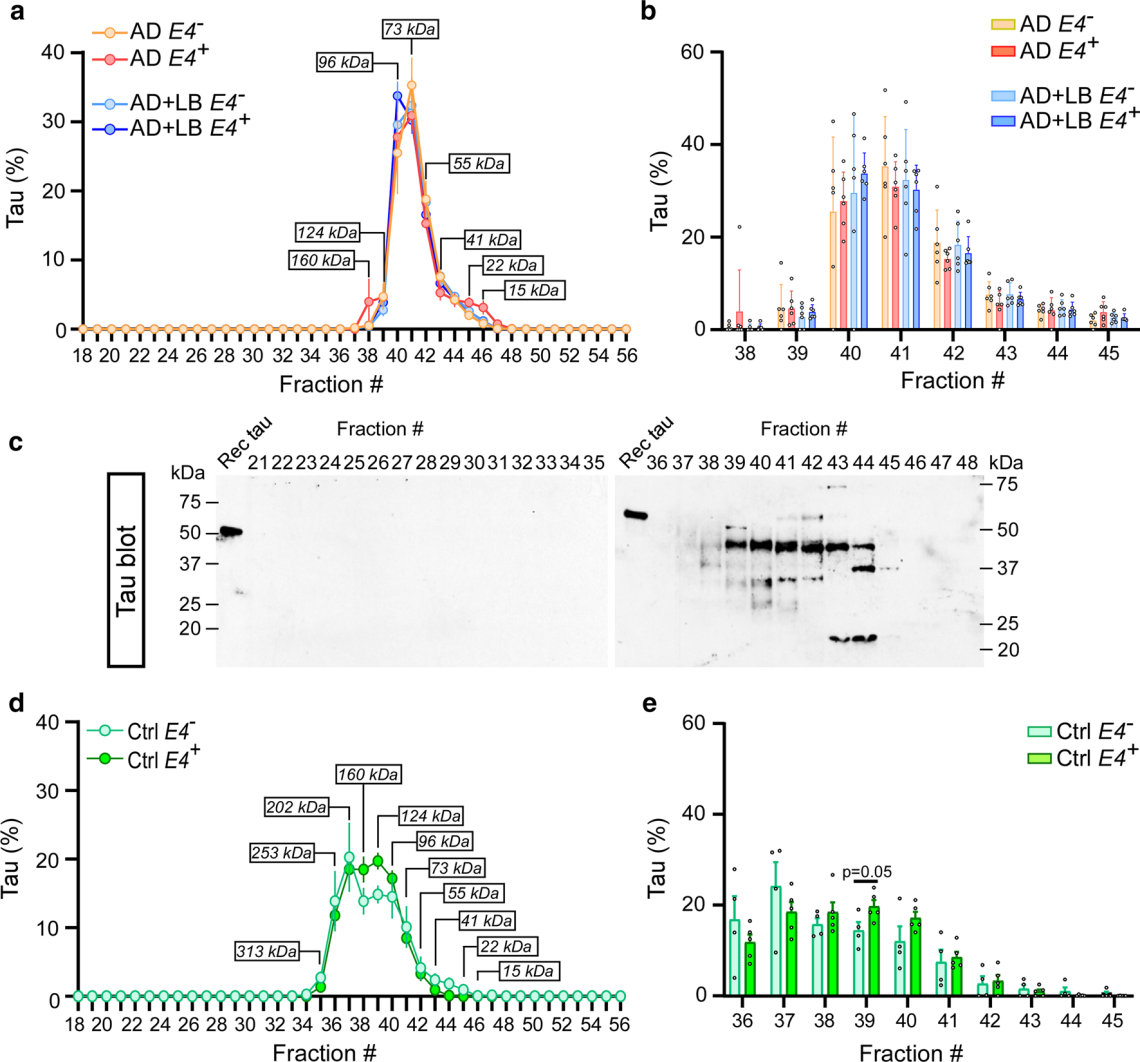
The size distribution of tau in TBS brain lysates from AD and control brains. TBS-soluble human brain lysates from AD, AD + LB, and control brains were fractioned by SEC as described in Fig. 3. The levels of tau were measured by ELISA (**a**, **b**, **d**, and **e**) and Western blotting (**c**). The size distribution of tau in the AD and AD + LB brains (**a–c**), control brains (**d** and **e**), without or with *APOE4* gene allele, were quantified and compared. *N* = 6 samples/group in AD cases. Two-way ANOVA with Sidak’s multiple comparison tests were used for statistical analyses (**b**). *N* = 4–5 samples/group in control cases. The student *t* tests were used for statistical analyses (e). Data are shown as mean ± SEM. Only the significantly changed fractions were labeled

We observed that the size distribution of soluble tau proteins was different in AD and control brains (Fig. 4a–e). In AD, about 60% of soluble tau proteins were in the size range of 73 and 96 kDa (fractions #40 and #41, Fig. 4a–c); whereas the peaks of tau proteins in control brains were distributed from 96 to 202 kDa (fractions #37 to #40, Fig. 4d–e). These findings imply potential conformational changes of soluble tau proteins in AD brains but would need further investigation (Fig. 4d and e). There were no significant differences in the size distribution of tau protein with or without LB co-pathology or in *APOE4* carriers and non-carriers from AD and control brains (Fig. 4b and e).

**Fig. 5:**
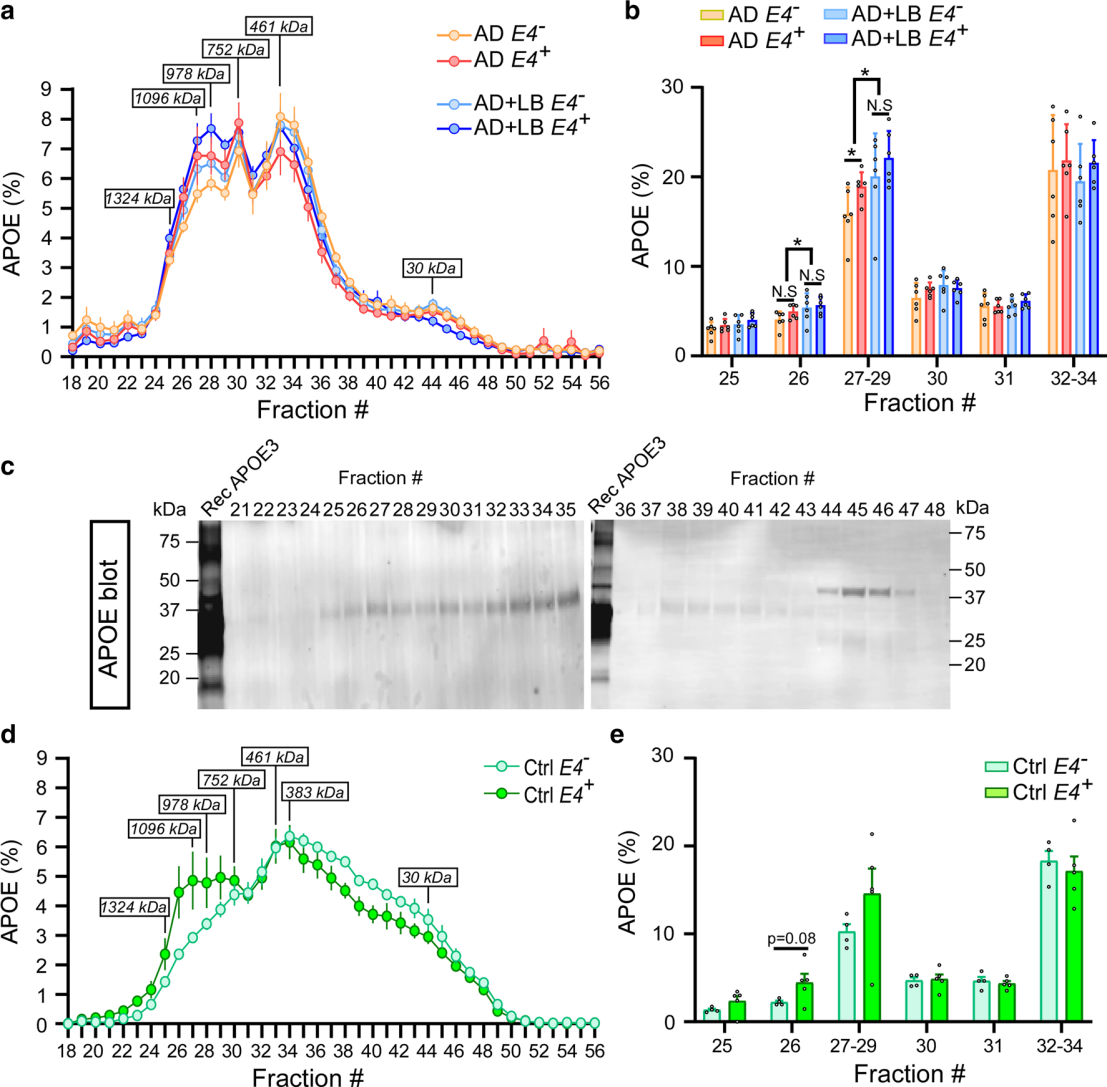
The size distribution of APOE in TBS brain lysates from AD and control brains. TBS-soluble human brain lysates from AD, AD + LB, and control brains were fractioned by SEC as described in Fig. 3. The levels of APOE were measured by ELISA (**a**, **b**, **d**, and **e**) and Western blotting (**c**). The size distribution of APOE in the AD and AD + LB brains (**a–c**), control brains (**d** and **e**), without or with *APOE4* gene allele, were quantified and compared. *N* = 6 samples/group in AD cases. Two-way ANOVA with Sidak’s multiple comparison tests were used for statistical analyses (**b**). *N* = 4–5 samples/group in control cases. The student *t* tests were used for statistical analyses (**e**). Data are shown as mean ± SEM. Only the significantly changed fractions were labeled

The peaks of APOE-containing fractions were identified at the size of 978 kDa, 752 kDa, and 461 kDa in AD (Fig. 5a and b). Interestingly, the presence of LB pathology significantly increased the percentage of large APOE proteins in fraction #26 and in peak fractions including #27, #28, and #29 (Fig. 5a and b). In AD cases without LB pathology, *APOE4* carriers had more large-sized APOE (fractions #27-#29) compared to *APOE4* non-carriers (Fig. 5a–c). In control brains, the major peak of soluble APOE proteins was at the size of 383 kDa (fraction #34) which is smaller than those AD brains (Fig. 5d). *APOE4* carriers showed a greater amount of larger size APOE protein (fraction #26 to #30) than *APOE4* non-carriers, although the difference was not significant due to large sample-to-sample variations (Fig. 5e). Altogether, these data suggest that the presence of AD and LB pathologies may modify the structure and/or aggregation of soluble APOE resulting in increased sizes. Moreover, APOE4 may contribute to disrupting the structure of both α-SYN and APOE proteins in AD.

We were not able to detect Aβ40 and Aβ42 in any of the FPLC fractions (data not shown), which is likely due to low levels of these proteins in this soluble TBS fraction [35].

### High molecular weight α-SYN shows stronger seeding activity

**Fig. 6:**
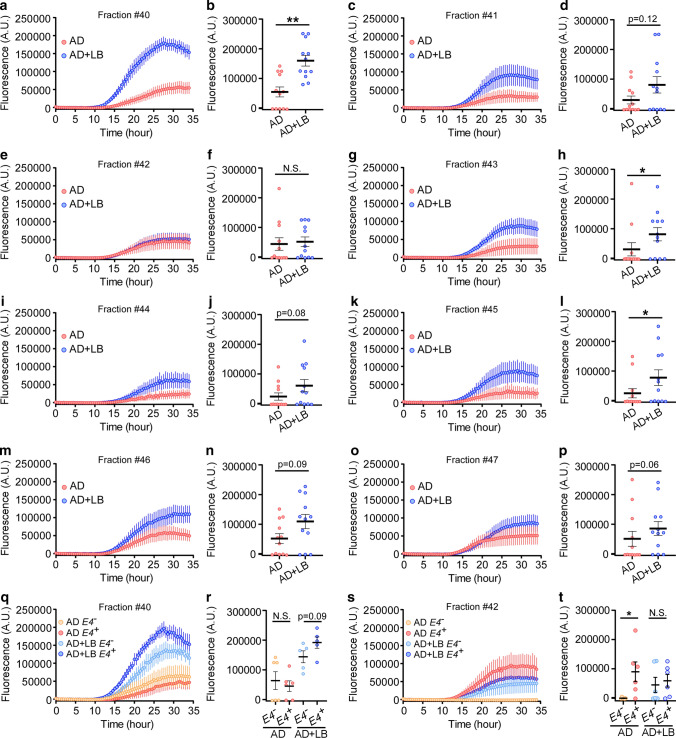
The contribution of different α-SYN species on the seeding activities. TBS-soluble human brain lysates from AD and AD + LB brains were fractioned by SEC as described in Fig. 3. The fractions from #40 to #47 containing α-SYN proteins were subjected to α-SYN RT-QuIC assays. (**a**, **c**, **e**, **g**, **i**, **k**, **m**, and **o**) The extent of aggregation was monitored by ThT fluorescence and the aggregation curves from fraction #40 to #47 are shown. (**b**, **d**, **f**, **h**, **j**, **l**, **n**, and **p**) The maximum fluorescence values (A.U., measured at plateau of aggregation) from fraction #40 to #47 are shown. Each dot represents the average value of an individual biological sample measured in duplicate. *N* = 11–12 samples/group. Data are mean ± SEM. The Mann–Whitney *U* tests were used for statistical analyses. *p < 0.05; **p < 0.01; *N.S.* not significant. **q**–**t** The α-SYN RT-QuIC aggregation curve (**q** and **s**) and the maximum fluorescence values (**r** and **t**) from the fraction #40 and #42 are shown by further separating *APOE* genotypes (without or with *APOE4* gene allele). *N* = 5–6 samples/group. Data are mean ± SEM. The Mann–Whitney *U* tests were used for statistical analyses. *p < 0.025; *N.S*. not significant

To further characterize the α-SYN seeds in TBS soluble fractions, we performed RT-QuIC assays on FPLC fractions containing α-SYN (fractions #40 to #47) in AD brains (Fig. 3a–c). Interestingly, fraction #40, with molecular weight ~ 96 kDa, had the highest ThT fluorescence intensity (with average fluorescence A.U. at ~ 200,000) in AD + LB, and this was significantly greater than in AD (Fig. 6a and b). The other fractions, #43 (Fig. 6g and h) and #45 (Fig. 6k and l), also showed significantly higher RT-QuIC signal in AD + LB than in AD. Similar trends were observed in fractions #41 (Fig. 6c and d), #44 (Fig. 6i and j), #46 (Fig. 6m and n), and #47 (Fig. 6o and p); however, the differences were not statistically significant. As expected, fraction #42, containing physiological forms of α-SYN, showed no significant difference in seeding activity between AD and AD + LB (Fig. 6e and f).

When comparing fraction #40 in *APOE4* carriers and non-carriers we found that AD + LB *APOE4*^+^ cases had the highest ThT fluorescence compared with AD + LB *APOE4*^*−*^ cases, but the difference was not significant (*p* = 0.09, Fig. 6q and r). In fraction #42, the AD *APOE4*^*−*^ cases had no RT-QuIC signal in any of the samples, further supporting the physiological nature of α-SYN in this molecular size fraction (Fig. 6s and t). Interestingly, the AD *APOE4*^+^ cases showed similar RT-QuIC signals as AD + LB cases, which were both significantly higher than AD *APOE4*^*−*^ cases. This may indicate that APOE4 disturbs the physiological properties of α-SYN in this fraction (Fig. 6s and t). Altogether, these results suggest that the α-SYN species with higher molecular weight might have the greatest contribution to α-SYN seeding activity in the presence of LB and *APOE4*.

### α-SYN aggregates derived from AD + LB brains are more resistant to proteolysis

**Fig. 7:**
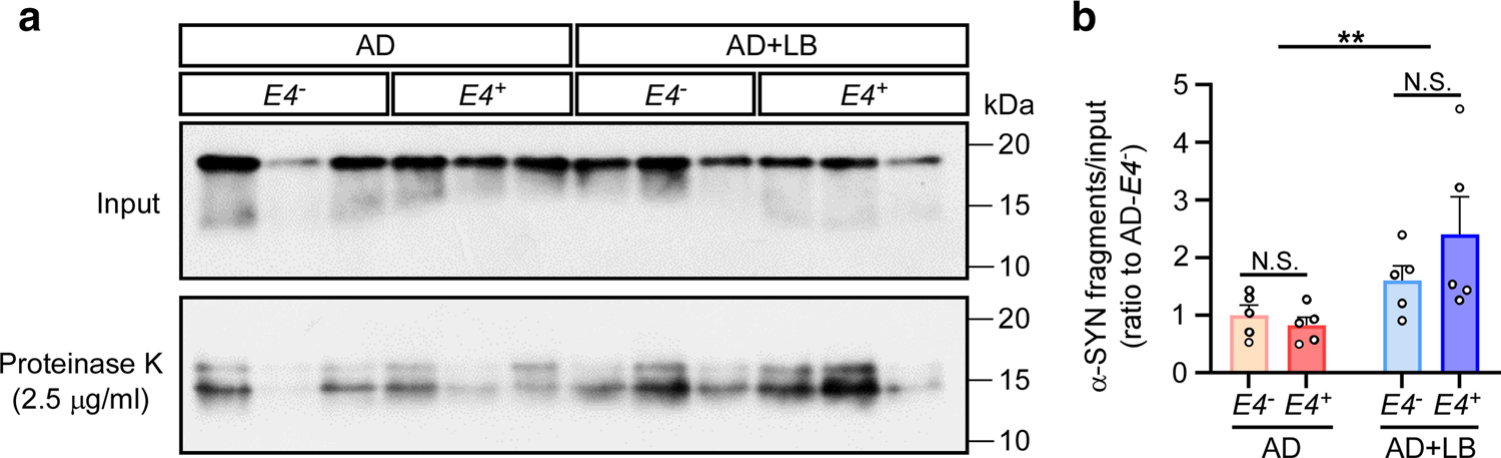
Protease resistance of α-SYN aggregates derived from the brains of patients with AD. α-SYN-RT-QuIC products derived from TBS lysates of brains from patients with AD (*APOE4*^*−*^, *APOE4*^+^) and AD + LB (*APOE4*^*−*^, *APOE4*^+^) were incubated without (input) or with 2.5 μg/ml of protease K at 37 °C for 30 min. Samples were then subjected to Western blotting to detect α-SYN (**a**). The ratio of the undigested α-SYN fragment and the total α-SYN (input) were quantified and compared among groups (**b**). Molecular weight markers (kDa) are indicated on the right of each blot. *N* = 5 samples per group. Two-way ANOVA with Sidak’s multiple comparison tests were used for statistical analyses. **p < 0.01; *N.S.* not significant

To further evaluate α-SYN aggregates in AD and AD + LB with different *APOE* genetic background, we examined their resistance to proteolytic degradation. The amplified α-SYN aggregates from RT-QuIC were incubated with proteinase K at 2.5 μg/ml. After 30 min of incubation, the aggregates were digested and bands with a molecular weight around 15 kDa remained as fragments from these samples (Fig. 7a). When analyzing the ratio of the fragments to total α-SYN input, we found that α-SYN aggregates from AD + LB had significantly more fragments compared to AD. This further implies that α-SYN aggregates in AD + LB are conformationally different from those in AD and, thus, are more resistant to proteinase K (Fig. 7a and b). We did not observe significant differences between *APOE4*^+^ and *APOE4*^*−*^ cases (Fig. 7a and b).

### α-SYN aggregates from AD + LB brains have higher seeding ability in α-SYN biosensor cells

**Fig. 8:**
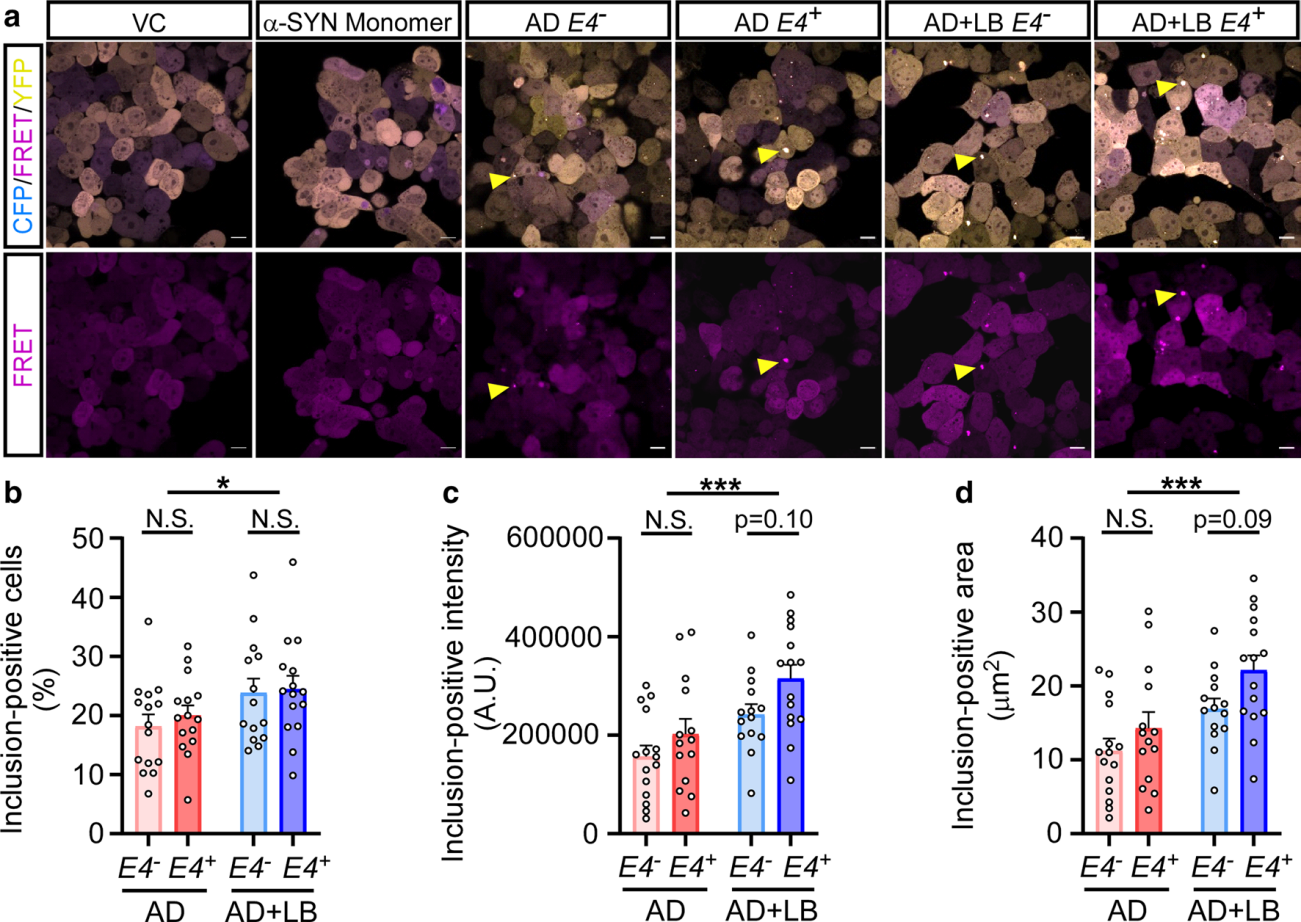
The seeding activity of α-SYN aggregates derived from human AD brain samples in α-SYN biosensor cells. The α-SYN biosensor cells expressing α-SYN (A53T) mutant protein fused to cyan and yellow fluorescent proteins (α-SYN-CFP/YFP) were treated with vehicle (vehicle control, VC), 0.35 μM α-SYN monomer, or 0.35 μM amplified α-SYN aggregates from the TBS brain lysates of patients with AD (*APOE4*^*−*^, *APOE4*^+^) or AD + LB (*APOE4*^*−*^, *APOE4*^+^). **a–d** After 72 h of incubation, cells were harvested and subjected to fluorescence resonance energy transfer (FRET)-based assay by fluorescence microscopy. Representative images of each experimental group are shown with a merged channel of CFP, FRET and YFP, and individual channel of FRET (**a**). Inclusions in FRET channel were subjected to quantification **b–d**, the yellow arrowhead shows the example of inclusion). The percentage of inclusion-positive cells (**b**), the intensity of the inclusions (**c**), and the size of inclusions (**d**) were quantified. Scale bars: 10 μm. Experiments were carried out with three independent experiments. Images from 4–5 fields per group were captured, each dot represents an individual field. Data are shown as mean ± SEM. Two-way ANOVA with Sidak’s multiple comparison tests were used for statistical analyses. *p < 0.05; ***p < 0.001; *N.S.* not significant

We next tested the seeding activity of these amplified α-SYN aggregates derived from AD brains using an in vitro cellular system. We performed a fluorescence resonance energy transfer (FRET)-based assay using α-SYN biosensor cells overexpressing α-SYN-A53T mutant protein which was fused to cyan and yellow fluorescent proteins (α-SYN-CFP/YFP) [17, 24, 64]. As previously reported, the exogenous α-SYN seeds can trigger the intracellular aggregation of the α-SYN in biosensor cells reflected by the FRET signals (CFP and YFP) [19, 24]. To test whether the amplified α-SYN from the AD brains can induce α-SYN seeding activity in cells, we incubated the α-SYN biosensor cells with 0.35 μM α-SYN aggregates from AD and AD + LB brains with different *APOE* genotypes. After 72 h of treatment, we observed FRET-positive α-SYN inclusions in these cells (Fig. 8a). The percentage of cells containing FRET-positive α-SYN inclusions were significantly higher in the AD + LB group compared to AD (Fig. 8b). The intensity and size of the FRET-positive inclusions were significantly different between AD + LB versus AD groups (Fig. 8c and d). Among them, the amplified α-SYN aggregates from AD + LB *APOE4*^+^ group showed the highest seeding activity (Fig. 8c and d). As controls, the cells treated with vehicle control (VC) and α-SYN monomer at the same concentration as the amplified aggregates (0.35 μM) did not generate any visible inclusions (Fig. 8a).

### α-SYN aggregates derived from AD + LB *APOE4*^+^ brains are toxic to neurons

**Fig. 9:**
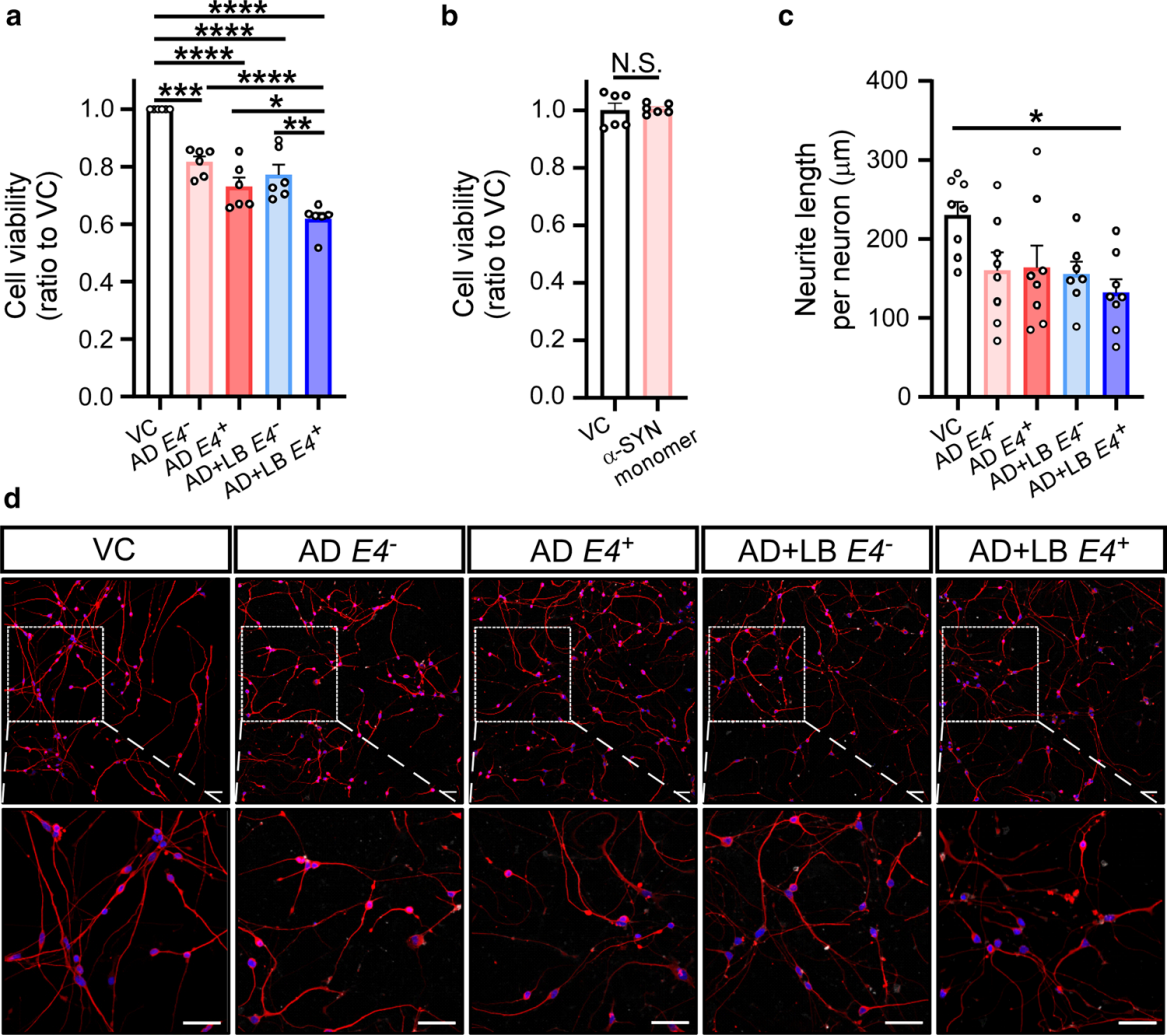
The cytotoxicities of α-SYN aggregates derived from human AD brain samples in iPSC-derived neurons. The human-induced pluripotent stem cells (iPSC)-derived neurons at DIV 12 were treated with vehicle (vehicle control, VC) or 0.35 μM amplified α-SYN aggregates from the TBS brain lysates of patients with AD (*APOE4*^*−*^, *APOE4*^+^) or AD + LB (*APOE4*^*−*^, *APOE4*^+^) (**a**), VC or 0.35 μM α-SYN monomers (**b**). After 48 h of incubation, cells were harvested and the cell viability was determined by MTT assay. Experiments were carried out in duplicate with three independent experiments, each dot represents an individual replicate. For the measurement of neurite length (**c** and **d**), the cells treated with VC or 0.35 μM amplified α-SYN aggregates were fixed and stained with TUJ1 antibody (red). The nuclei were stained with DAPI (blue). The average length of TUJ1 positive neurites per nuclei were quantified (**c**). Representative images of each experimental group are shown (**d**). Scale bars: 50 μm. Experiments were carried out with three independent experiments. Images from 2 to 3 fields per group were captured, each dot represents an individual field. Data are shown as mean ± SEM. One-way ANOVA with Holm-Sidak’s multiple comparison tests were used for statistical analyses in panel **a** and **c**. The student *t* test was used for statistical analysis in panel **b**. *p < 0.05; **p < 0.01; ***p < 0.001; ****p < 0.0001; *N.S.* not significant

Finally, we tested the neurotoxicity of these α-SYN aggregates from AD brains using human iPSC-derived neurons. The iPSCs were generated and characterized from fibroblasts obtained from a healthy individual as previously described [69]. After 14 days of differentiation, these iPSC-derived neurons expressed neuronal markers including MAP2, TUJ1, and the glutamatergic marker vGLUT1, but not GABAergic marker GAD67 or dopaminergic marker TH, suggesting that these neurons were mostly glutamatergic neurons, which is consistent with the previous report using the similar differentiation protocol (Supplementary Fig. 3, online resource) [16]. Next, we tested cytotoxicity by incubating neurons with α-SYN aggregates from AD and AD + LB with different *APOE* genotypes. We found that α-SYN aggregates, at a concentration of 0.35 μM, showed toxicity in all the samples compared with vehicle control (Fig. 9a), whereas the same amount of α-SYN monomer treatment did not generate any cytotoxicity in neurons (Fig. 9b). Importantly, the α-SYN aggregates from AD + LB *APOE4*^+^ had the highest toxicity in neurons compared to aggregates from AD *APOE4*^*−*^, AD *APOE4*^+^, and AD + LB *APOE4*^*−*^ (Fig. 9a). Furthermore, iPSC-derived neurons showed significantly shorter neurite length after treatment with α-SYN aggregates from AD + LB *APOE4*^+^ compared to vehicle control (Fig. 9c and d). This effect was not observed with α-SYN aggregates from other groups. Collectively, these results suggest that APOE4 may exacerbate the toxicity of α-SYN aggregates in AD.

## Discussion

In this study, we investigated the effects of the *APOE4* allele on α-SYN level and seeding activity in AD. We found significant positive correlations between α-SYN and other AD-related markers in cases with LB pathology. In particular, we found a strong positive correlation between insoluble α-SYN and insoluble Aβ40, Aβ42, tau and APOE. Importantly, we found that APOE4 exacerbates α-SYN seeding ability and related toxicities. Our findings support the hypothesis that LB pathology intensifies AD-type pathologies, providing mechanistic insight into how APOE4 impacts α-SYN pathogenesis in AD.

α-SYN, encoded by the *SNCA* gene, is a hydrophilic protein involved in presynaptic vesicle recycling and docking to facilitate neurotransmitter release [9, 46]. There is no universal agreement on the physiological properties of α-SYN. Some groups have demonstrated that the 14 kDa protein of α-SYN has no secondary structure in an aqueous solution, but rather exists as random coils. Nonetheless, α-SYN has a propensity to transform to a β-sheet conformation, driving neurodegeneration [1, 2, 5, 9, 15, 33, 44]. Furthermore, a recent study lends further support for this hypothesis as well as indicating that physiological α-SYN monomers are amino-terminally acetylated, thereby adopting a compact conformation that protects α-SYN from aggregation [58]. Conversely, in 2011 two separate groups reported that the predominant physiological species of α-SYN is a helically folded tetramer and that this tetrameric complex of α-SYN is resistant to aggregation [3, 62]. Our results with detergent-free TBS lysates revealed that the peak fraction of soluble α-SYN (around 40–50% of total α-SYN) in AD was at a molecular weight ~ 55 kDa, consistent with the size of tetramers [3]. Around 20% of α-SYN had a molecular weight larger than 55 kDa, while the remaining species were smaller than a tetramer. Our data indicate that at least in AD, there is the heterogeneity of α-SYN species in soluble lysates. A major physiologic role of α-SYN is as a lipid-binding protein in synaptic vesicle docking [37, 39], which is facilitated by adopting an α-helical structure upon binding to phospholipids [28]. Therefore, we cannot distinguish whether the 55 kDa of α-SYN peak is the lipidated α-SYN complex or α-SYN tetramer. Further studies examining the structure of α-SYN and/or its lipid-association status are needed to further address these possibilities.

The development of α-SYN RT-QuIC as a tool for detecting seeding properties of the brain or tissue-derived α-SYN assesses template-seeded aggregation of recombinant monomeric α-SYN into amyloid fibrils [54]. We show that high molecular weight soluble α-SYN species (~ 96 kDa) have the strongest seeding activity and that they are associated with LB pathology, while lower molecular weight α-SYN species (55 kDa) have the lowest seeding activity among all α-SYN species. These findings support the notion that α-SYN species at 55 kDa might be physiological, but not pathogenic species [3]. Nonetheless, future investigations are needed to better elucidate the structure of high molecular weight, likely pathogenic, α-SYN. Further, our findings show that the α-SYN aggregation rate (PAR) in RT-QuIC is positively correlated with the insoluble α-SYN levels, suggesting that the RT-QuIC assay can be applied to both disease diagnosis and severity evaluation due to its quantitative potential. However, the quantitative value of RT-QuIC assay in different disease conditions remains to be established.

*APOE4* is the common strong genetic risk factor for both AD and LBD. *APOE4* has been reported to be overrepresented in patients with AD + LB and “pure” LBD [8, 18, 59, 60]. The clinical phenotypes of LBD, such as cognitive function and survival rate, are modified by *APOE4* gene allele [55]. *APOE4* has also been implicated in cognitive impairment or motor dysfunction within Parkinson’s disease (PD) [11, 25, 26, 31, 36, 48, 50, 56]. These findings indicate that APOE4 may potentially accelerate α-SYN aggregation and spreading during the LB development. Our group has previously reported that *APOE4* increases LB pathological burden in postmortem human brains even when AD pathology is minimal [13]. In this study, we observed that the presence of *APOE4* gene allele exacerbates α-SYN seeding both in a large AD cohort and a smaller cohort of “pure” LBD cases, with the amount of APOE aggregates associated with α-SYN seeding activity in AD brains. Altogether, these findings imply that APOE impacts α-SYN seeding which might be independent of amyloid pathways, although this needs to be further validated using a larger cohort of LBD brains with minimal AD pathology. Additionally, we observed reduced membrane-associated α-SYN in AD + LB brains which was further decreased in *APOE4* carriers. This implies that the AD + LB brains, in particular *APOE4*^+^ cases, may have more severe neurodegeneration and synaptic loss compared with other groups; however, this was not assessed in this study and should be evaluated in the future investigation. Furthermore, the amplified α-SYN aggregates from AD + LB *APOE4*^+^ brains had the most neurotoxicity. Altogether, our findings imply that APOE plays an important role in modifying α-SYN seeding and toxicity.

The mechanism of how APOE influences α-SYN seeding is not clear. A recent report shows that α-SYN isolated from CSF of PD was bound to apolipoproteins, specifically APOE, such that when APOE was immuno-depleted, α-SYN levels were dramatically reduced in CSF of both PD and control cases [49]. These data are indicative of an interaction between α-SYN and APOE; however, we did not observe overlapping peaks between α-SYN and APOE in SEC fractionated brain lysates. We speculate that the presence of APOE aggregates – in particular from APOE4 – may modulate the conformation of α-SYN leading to higher seeding and greater neurotoxicity. Whether this effect is through direct interaction between APOE and α-SYN is not clear and needs further studies. Given the fact that APOE4 is associated with the risk of DLB and PDD, but not necessarily PD [6, 27], it is tempting to speculate that effects of APOE4 may manifest more in neocortex where AD and LBD pathologies are found, whereas it has minimal impact on subcortical or brainstem structures where Lewy pathology is predominant in PD.

Lastly, the α-SYN seeding activity in AD brains has not been reported using RT-QuIC assay. Our findings show that a subset of AD brains without visible LB pathology has positive α-SYN seeding activity. This observation has the caveat that the brain tissue used for histologic evaluation is not the exact same tissue used for the assessment of seeding activity. Additionally, despite having a high specificity, the RT-QuIC false-positive signal cannot be absolutely ruled out from the analysis. Nevertheless, this observation suggests a possibility that there may be a seed-competent form of α-SYN not associated with observable LB pathology. If this is the case, it is possible that these individuals have resilience factors that prevent abnormal α-SYN from forming LB. It is shown that LB formation involves a complex interplay between α-SYN fibrillization, posttranslational modifications, and interactions between α-SYN aggregates and membranous organelles, including mitochondria, the autophagosome, and endolysosome [38]. Another possibility is that the RT-QuIC assay is sensitive enough to detect α-SYN seeding ability and conformational changes even before the formation of LB. Further investigations with these individuals showing α-SYN seeding activity, with or without LB formation, may help to better understand the pathomechanisms of α-SYN aggregation in AD and LBD.

In summary, our study defines the level, solubility, and size of α-SYN, and its seeding activity in AD brains with or without LB co-pathologies, using a large human postmortem cohort. We also take a step forward translating how APOE4 affects α-SYN pathological aggregation and toxicity in AD patients. Our findings suggest an interplay between α-SYN and APOE4 in AD pathogenic cascade, providing insight into targeting α-SYN seeding in AD patients, in particular *APOE4* carriers.

## Supplementary Information

Below is the link to the electronic supplementary material.

Supplementary file1 (DOCX 3245 KB)
