# Supplementary material for: APOE4 exacerbates α-synuclein seeding activity and contributes to neurotoxicity in Alzheimer’s disease with Lewy body pathology
Source: Acta Neuropathol. 2022 Apr 26;143(6):641–62. doi: 10.1007/s00401-022-02421-8 (PMC9107450; doi:10.1007/s00401-022-02421-8)
Supplement: Supplementary file 1 — Supplementary file1 (DOCX 3245 KB) [file 401_2022_2421_MOESM1_ESM.docx]

**Supplementary materials**

**APOE4 exacerbates α-synuclein seeding activity and contributes to neurotoxicity in Alzheimer’s disease with Lewy body pathology**

Jin et al., 2022

**
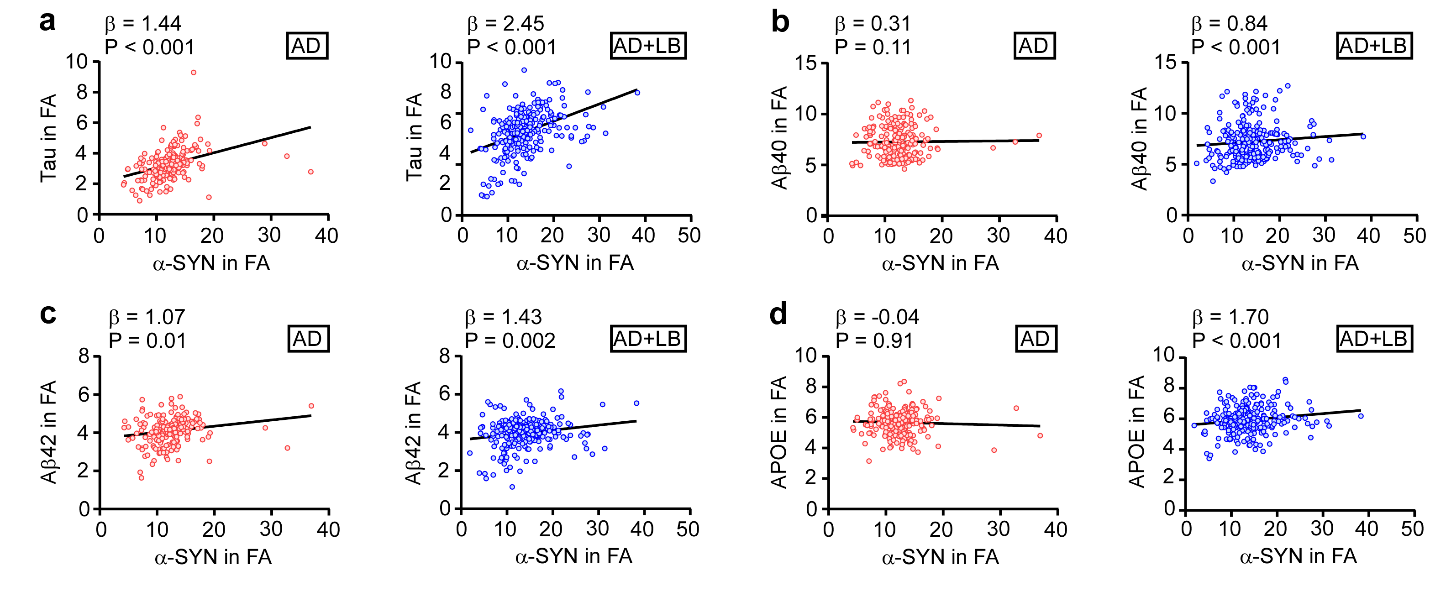
**

**Supplementary Fig. 1** **Scatterplots showing the correlations between insoluble α-SYN and insoluble tau, Aβ40, Aβ42, and APOE**

The correlations between the levels of α-SYN and those of Tau (**a**), Aβ40 (**b**), Aβ42 (**c**), and APOE (**d**) in the formic acid (FA) brain lysates from patients with AD and AD+LB are shown as scatterplots. The correlation coefficent (β) and p-value were calculated using linear regression models that were adjusted for age at death, sex, CAA score, Braak stage, Thal phase, and the number of *APOE4* alleles. The α-SYN and tau levels were examined on the square root scale; and Aβ40, Aβ42, and APOE levesl were examined on the natural logarithm scale. N = 214 and 255 cases in AD and AD+LB groups, respectively.

**
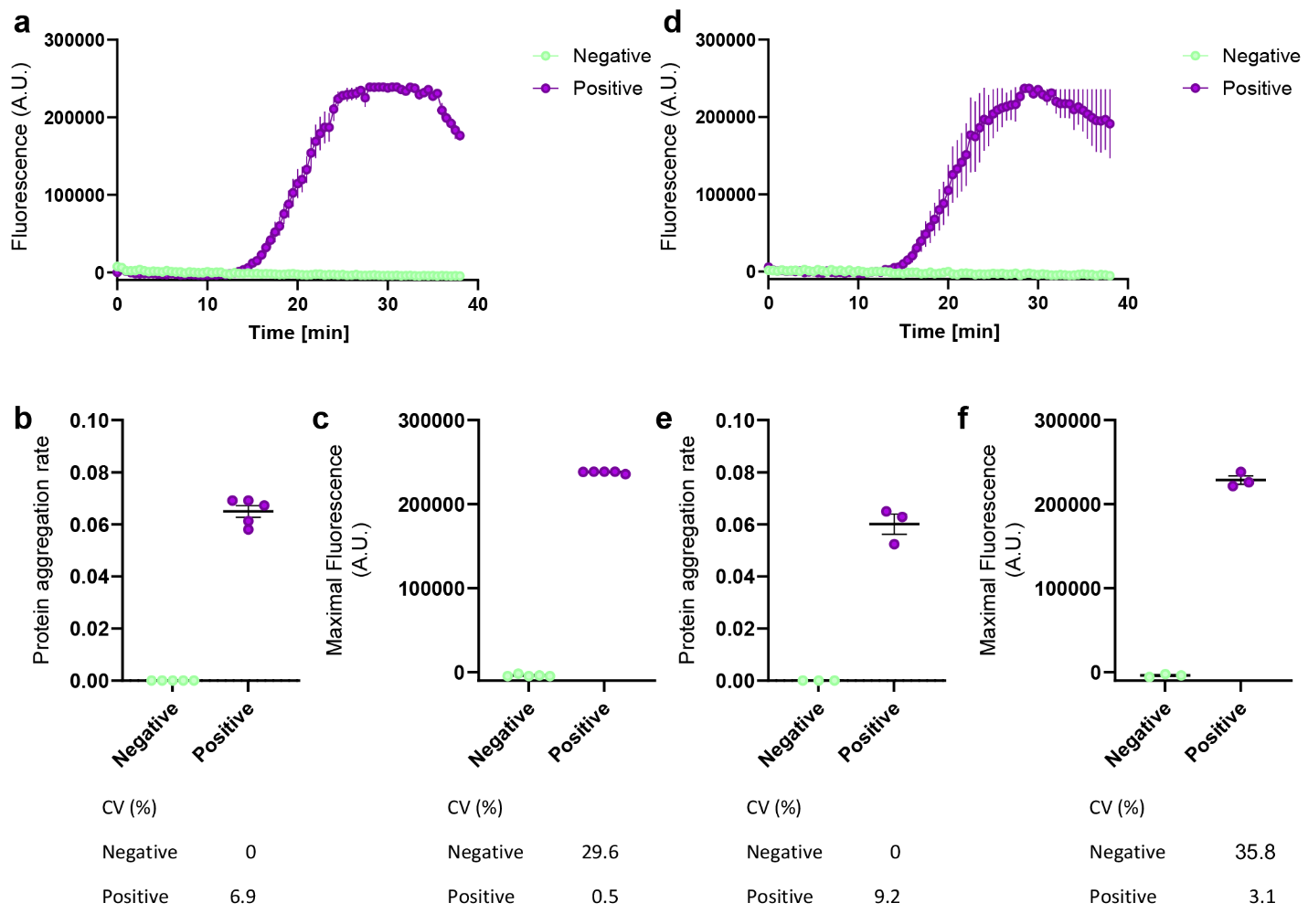
**

**Supplementary Fig. 2 Intra- and inter-batch variation of RT-QuIC assay**

Samples of TBS brain lysates from normal control brain (negative) and LBD (positive) brain were subjected to α-SYN RT-QuIC assay with 5 replicate/group/batch for three different batches in total**. (a and d)** The aggregation curves are shown (data are mean ± SEM). The Intra-batch (**a-c**) and inter-batch (**d-f**) percentage of coefficent of variations (CV%) were calculated based on the PAR (**b and e**) and maximum fluorescence values (A.U., measured at plateau of aggregation, **c and f**). The intra-batch CV% was calculated with 5 replicates/group and the inter-batch CV% was calucated with 3 batches/group. (**b, c, e, and f**) Each dot represents the value of an individual biological sample.

**
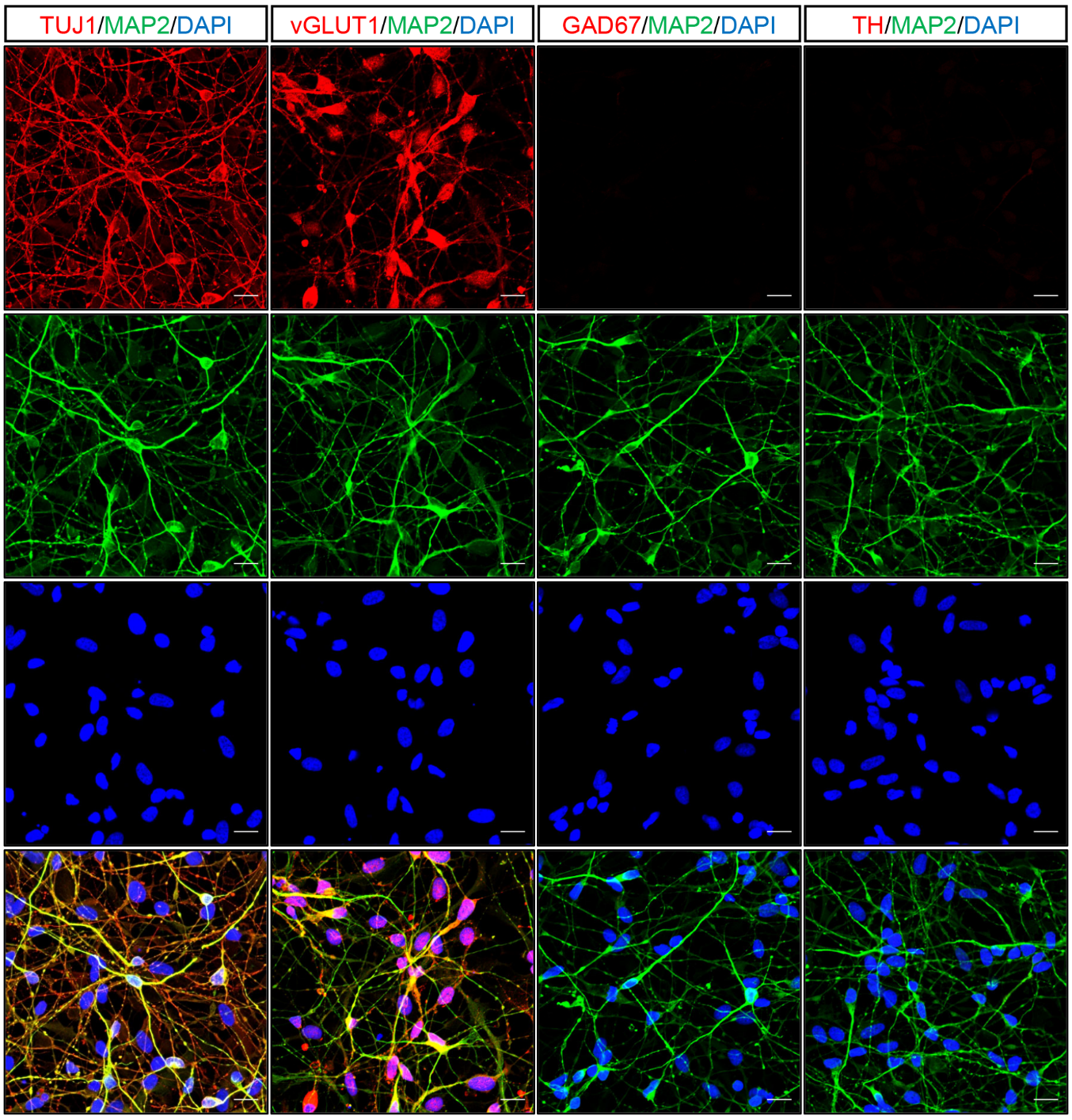
**

**Supplementary Fig. 3** **Characterization of iPSC-derived neurons**

The human induced pluripotent stem cells (iPSC)-derived neurons at DIV 14 were immuno-stained with different neuronal markers, including MAP2, TUJ1, vGLUT1, GAD67, and TH. Nuclei were visualized with DAPI. Representative images are shown. Scale bars: 20 μm.

**Supplementary Table 1 Overview of patient characteristics.**

| **Variable** | **Total N** | **Median (minimum, maximum) or No. (%)** |
| --- | --- | --- |
| **Age (years)** | 469 | 82 (55, 100) |
| **Sex (Male)** | 469 | 224 (47.8%) |
| ***APOE* genotype** | 469 |  |
| **E2/E3** |  | 6 (1.3%) |
| **E2/E4** |  | 14 (3.0%) |
| **E3/E3** |  | 152 (32.4%) |
| **E3/E4** |  | 230 (49.0%) |
| **E4/E4** |  | 67 (14.3%) |
| **Thal phase** | 468 |  |
| **2** |  | 3 (0.6%) |
| **3** |  | 32 (6.8%) |
| **4** |  | 37 (7.9%) |
| **5** |  | 396 (84.6%) |
| **Braak stage** | 469 |  |
| **IV** |  | 71 (15.1%) |
| **V** |  | 143 (30.5%) |
| **VI** |  | 255 (54.4%) |
| **CAA score** | 469 | 0.7 (0.0, 4.0) |
| **Co-LB pathology** | 469 |  |
| **AD** |  | 214 (45.6%) |
| **AD + ALB** |  | 75 (16.0%) |
| **AD + BLBD** |  | 16 (3.4%) |
| **AD + TLBD** |  | 79 (16.8%) |
| **AD + DLBD** |  | 85 (18.1%) |
| **α-SYN TBS** | 466 | 661.9 (44.5, 1779.6) |
| **α-SYN TBSX** | 467 | 325.1 (36.1, 1933.4) |
| **α-SYN FA** | 466 | 161.2 (3.6, 1466.3) |
| **Total tau TBS** | 469 | 4.3 (0.0, 26.3) |
| **Total tau TBSX** | 469 | 1340.9 (63.1, 4272.5) |
| **Total tau FA** | 466 | 10.9 (0.4, 86.4) |
| **Aβ40 TBS** | 469 | 66.8 (0.1, 8212.2) |
| **Aβ40 TBSX** | 469 | 261.7 (8.2, 17365.9) |
| **Aβ40 FA** | 469 | 1070.1 (28.3, 335472.8) |
| **Aβ42 TBS** | 469 | 628.0 (143.2, 9741.5) |
| **Aβ42 TBSX** | 469 | 1394.1 (235.0, 15364.7) |
| **Aβ42 FA** | 469 | 61.4 (3.2, 510.0) |
| **APOE TBS** | 468 | 461.9 (7.8, 1520.6) |
| **APOE TBSX** | 467 | 235.1 (33.8, 1027.7) |
| **APOE FA** | 469 | 336.5 (23.3, 5256.9) |
| **Superior temporal LB count** | 129 | 10 (0, 33) |

**Supplementary Table 2 Patient characteristics of AD cases used for RT-QuIC assay.**

|  | **AD without LB pathology**  **(AD, N=43)** | | **AD with LB pathology**  **(AD+LB, N=47)** | |
| --- | --- | --- | --- | --- |
| **Variable** | ***APOE4* non-carrier (N=22)** | ***APOE4* carrier**  **(N=21)** | ***APOE4* non-carrier (N=23)** | ***APOE4* carrier**  **(N=24)** |
| **Age (years)** | 84.5 (55.0, 93.0) | 85.0 (61.0, 95.0) | 81.0 (57.0, 96.0) | 78.0 (60.0, 89.0) |
| **Sex (Male)** | 11 (50.0%) | 10 (47.6%) | 11 (47.8%) | 12 (50.0%) |
| **Thal phase** |  |  |  |  |
| **2** | 1 (4.5%) | 0 (0.0%) | 0 (0.0%) | 0 (0.0%) |
| **3** | 3 (13.6%) | 1 (4.8%) | 4 (17.4%) | 1 (4.2%) |
| **4** | 2 (9.1%) | 5 (23.8%) | 2 (8.7%) | 7 (29.2%) |
| **5** | 16 (72.7%) | 15 (71.4%) | 17 (73.9%) | 16 (66.7%) |
| **Braak stage** |  |  |  |  |
| **IV** | 3 (13.6%) | 6 (28.6%) | 7 (30.4%) | 9 (37.5%) |
| **V** | 11 (50.0%) | 7 (33.3%) | 11 (47.8%) | 4 (16.7%) |
| **VI** | 8 (36.4%) | 8 (38.1%) | 5 (21.7%) | 11 (45.8%) |

The sample median (minimum, maximum) is given for age.

**Supplementary Table 3 Transformations of measures that were utilized in linear regression analysis.**

| **Outcome measure** | **Transformation** |
| --- | --- |
| **α-SYN TBS** | Square root |
| **α-SYN TBSX** | Square root |
| **α-SYN FA** | Square root |
| **CAA score** | Square root |
| **APOE TBS** | Square root |
| **APOE TBSX** | Square root |
| **APOE FA** | Natural logarithm |
| **Aβ40 TBS** | Natural logarithm |
| **Aβ40 TBSX** | Natural logarithm |
| **Aβ40 FA** | Natural logarithm |
| **Aβ42 TBS** | Natural logarithm |
| **Aβ42 TBSX** | Natural logarithm |
| **Aβ42 FA** | Natural logarithm |
| **Total tau TBS** | Square root |
| **Total tau TBSX** | Square root |
| **Total tau FA** | Square root |
| **Superior temporal LBs** | Square root |
| **Protein aggregation rate of RT-QuIC** | Converted to an ordinal variable:  low threshold: < 0.046  median threshold: >= 0.046 & < 0.053  high threshold: >= 0.053 |
| **Maximum fluorescence of RT-QuIC** | Converted to an ordinal variable:  no seeding activity: < 3063.85  low seeding activity: >= 3063.85 & < 126966.37  median seeding activity: >= 126966.37 & < 187940.60  and high seeding activity: >= 187940.60 |
